# Evidence for a role of RUNX1 as recombinase cofactor for TCRβ rearrangements and pathological deletions in ETV6-RUNX1 ALL

**DOI:** 10.1038/s41598-020-65744-0

**Published:** 2020-06-22

**Authors:** V. Seitz, K. Kleo, A. Dröge, S. Schaper, S. Elezkurtaj, N. Bedjaoui, L. Dimitrova, A. Sommerfeld, E. Berg, E. von der Wall, U. Müller, M. Joosten, D. Lenze, M. M. Heimesaat, C. Baldus, C. Zinser, A. Cieslak, E. Macintyre, C. Stocking, S. Hennig, M. Hummel

**Affiliations:** 1 Charité University Medicine Berlin, Institute of Pathology, Berlin, Germany; 2 HS Diagnomics GmbH, Berlin, Germany; 3 University of Paris, Institute Necker-Enfants Malades (INEM), INSERM U1151, Laboratoire d’Onco-Hematology, Assistance Publique-Hôpitaux de Paris (AP-HP), Hôpital Necker Enfants-Malades, Paris, France; 4 Heinrich-Pette-Institute, Leibniz-Institute for Experimental Virology, Hamburg, Germany; 5 Charité University Medicine Berlin, Institute of Microbiology, Infectious Diseases and Immunology, Berlin, Germany; 6 University Medical Center Schleswig-Holstein, Department of Internal Medicine II, Kiel, Germany; 7 Precigen Bioinformatics Germany GmbH, Munich, Germany; 8 University Medical Center Eppendorf, Department of Stem Cell Transplantation, Hamburg, Germany

**Keywords:** Cancer, Evolution, T-cell receptor, Molecular biology

## Abstract

T-cell receptor gene beta (TCRβ) gene rearrangement represents a complex, tightly regulated molecular mechanism involving excision, deletion and recombination of DNA during T-cell development. RUNX1, a well-known transcription factor for T-cell differentiation, has recently been described to act in addition as a recombinase cofactor for TCRδ gene rearrangements. In this work we employed a RUNX1 knock-out mouse model and demonstrate by deep TCRβ sequencing, immunostaining and chromatin immunoprecipitation that RUNX1 binds to the initiation site of TCRβ rearrangement and its homozygous inactivation induces severe structural changes of the rearranged TCRβ gene, whereas heterozygous inactivation has almost no impact. To compare the mouse model results to the situation in Acute Lymphoblastic Leukemia (ALL) we analyzed TCRβ gene rearrangements in T-ALL samples harboring heterozygous Runx1 mutations. Comparable to the *Runx1*^+/−^ mouse model, heterozygous Runx1 mutations in T-ALL patients displayed no detectable impact on TCRβ rearrangements. Furthermore, we reanalyzed published sequence data from recurrent deletion borders of ALL patients carrying an *ETV6-RUNX1* translocation. RUNX1 motifs were significantly overrepresented at the deletion ends arguing for a role of RUNX1 in the deletion mechanism. Collectively, our data imply a role of RUNX1 as recombinase cofactor for both physiological and aberrant deletions.

## Introduction

RUNX1 belongs to the evolutionary conserved Runt transcription factor family and is indispensable for the establishment of definitive hematopoiesis in vertebrates and is an important regulator of cells of the immune system^1–3^. *RUNX1* is among the most frequently mutated genes in various hematological malignancies and *RUNX1* alterations can lead to a loss of RUNX1 function or to a dominant-negative effect^4,5^. Mono-allelic *RUNX1* mutations occur in approximately 15% of T-Cell Acute Lymphoblastic Leukemia (T-ALL), predominantly in cases with an immature phenotype and a poor prognosis^6–8^. In *de novo* Acute Myeloid Leukemia (AML) patients, somatic mutations in *RUNX1* are detectable in approximately 3% of children and 15% of adults^4^. In an AML subgroup with an immature phenotype (AML-M0), 30% of the cases are associated with bi-allelic inactivating *RUNX1* point mutations and deletions^9^. Patients with Myelodysplastic Syndrome (MDS) carrying *RUNX1* mutations have a higher risk and shorter latency for progression to AML^10^.

Furthermore, there are over 50 different types of chromosomal translocations affecting *RUNX1*^4^. We focus in this study on the most common translocation, t(12;21), occurring in approximately 20% of childhood B-cell precursor (BCP) ALL patients^11,12^. This translocation results in an *ETV6-RUNX1* fusion gene, encoding the N-terminal non-DNA binding moiety of *ETV6* (12p13) fused to the almost entire RUNX1 protein coding region (21q22) including its DNA-binding Runt-domain (RHD), transactivation domain (TAD) and the VWRPY motif^4^. Twin studies have shown that the *ETV6-RUNX1* translocation is the founder translocation in this BCP-ALL subgroup and is acquired *in utero* in very early progenitor cells prior to T- or B-cell receptor gene rearrangements^12,13^. Further genetic alterations leading to ALL can develop after years of latency^13^. Papaemmanuil and colleagues have characterized secondary events associated with leukemic transformation in *ETV6-RUNX1* ALL, employing exome and low-coverage whole-genome sequencing. They found an enrichment of binding sites for the recombination activating gene (RAG) proteins in close vicinity to the genomic breakpoint junctions and concluded that RAG-mediated recombination is the predominant driver of oncogenic rearrangement in *ETV6-RUNX1* ALL^14^.

**Figure 1:**
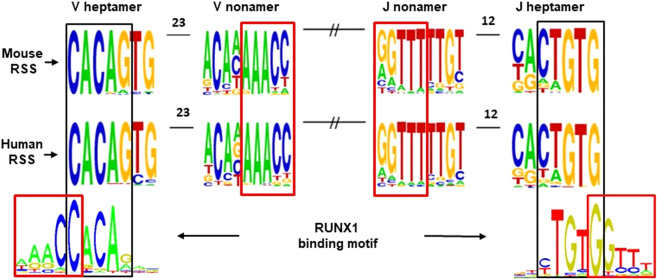
Overlap between RSS motifs and RUNX1 binding sites. IMGT murine and human RSS consensus motif logos (http://www.imgt.org/IMGTrepertoire/LocusGenes) generated from the 7 IG and TCR loci are shown^54^. The motif overlap between the RUNX1 binding core motif (http://jaspar.genereg.net) and the heptamer and nonamer motifs is marked with black and red squares, respectively. An overlap of the RUNX1 and the heptamer motif was previously reported for the human TCRδ D2 segment^18^.

The biological role of RAG proteins is to generate Immunoglobulin (IG) and T-cell receptor (TCR) rearrangements^15^. Thereby functional IG or TCR receptors are assembled from preexisting sets of Variable (V), Joining (J) and — in case of TCRβ, TCRδ and IGH — from additional Diversity (D) gene segments^16^. These segments are flanked by recombination signal sequences (RSS) composed of conserved heptamer and nonamer sequences separated by a spacer of 12 or 23 base pairs (Fig. 1). Upon binding of RAG1/2 to the RSS, the DNA sequences between the recombined V(D)J segments are excised. During recombination the ends of V, D and J segments are frequently truncated and non-templated sequences (N nucleotides) are incorporated at the junctions^16,17^.

The role of the ETV6-RUNX1 fusion protein was hitherto almost exclusively linked to the role of RUNX1 as a transcription factor investigated in more than 3000 publications. However, the RUNX1 DNA-binding core motif TGTGGNNN overlaps with the RSS heptamer and nonamer motifs which are crucial for the recombination of IG and TCR gene segments (Fig. 1). This raises the possibility that RUNX1 might also act as a recombinase cofactor for both physiological and non-physiological deletions.

A role of RUNX1 as a recombinase cofactor in TCR rearrangements has been demonstrated by its binding to the human TCRδ D2 RUNX1 heptamer motif and by subsequent enhanced deposition of RAG1^18^. In addition direct interaction of RUNX1 and RAG1 was shown in the Molt-4 T-lymphoblastic cell line and in CD34 positive thymocytes^18^.

Our experiments were devised to confirm the recombinase cofactor function of RUNX1 and to expand its role for appropriate TCRβ rearrangements. To this end we analyzed in depth the outcome of TCRβ gene rearrangements in a *Runx1* knockout mouse model. Our results imply that RUNX1 functions as a recombinase cofactor in physiological deletion processes during antigen receptor rearrangements. In addition we provide for the first time evidence for aberrant recombinase activity of RUNX1 leading to genomic deletions in hematological malignancies. We propose a synergistic dual role of RUNX1 as both a transcription factor and a recombinase cofactor.

## Results

### RUNX1 knockout leads to a reduction of B- and T-cells and thymus atrophy

Functional TCR or IG rearrangements are essential for the survival of mature B- and T-cells^19^. To determine the role of RUNX1 in this process we assessed the histological characteristics of thymus and spleen tissues in wildtype, *Runx1*^+/−^ and *Runx1*^−/−^ mice (N = 10, each) and evaluated the number of B- and T-cells by immunohistochemistry (IHC). The characteristics of the mice used have been previously described (See Materials and Methods).

**Figure 2:**
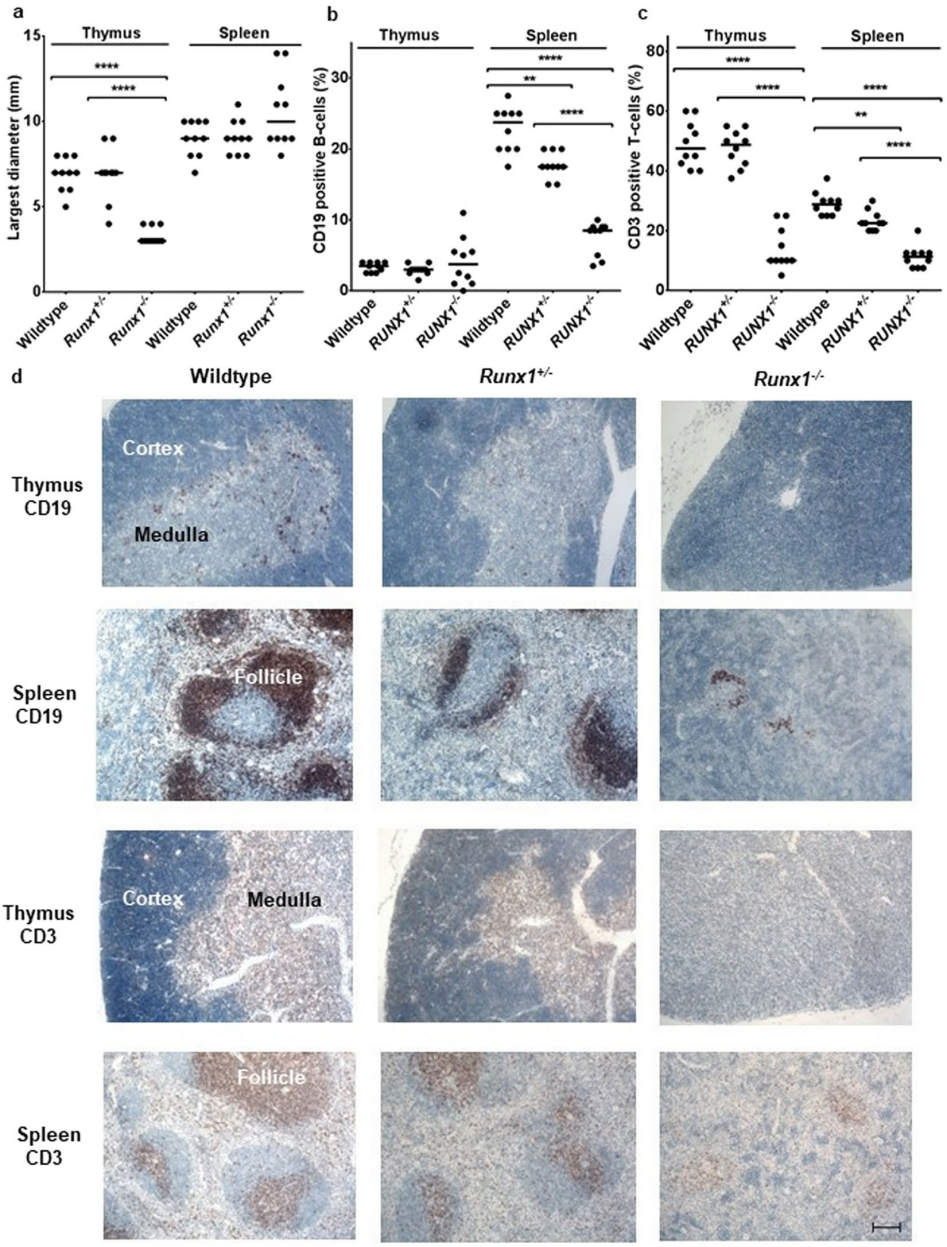
RUNX1 knockout leads to a reduction of B- and T-cells and thymus atrophy. (**a**) Size distribution (largest diameter) of spleens and thymi. **(b)** Percentage of CD19 positive B-cells and **(c)** Percentage of CD3 positive T-cells in thymi and spleens. Median values for each group are indicated (**P < 0.002, ****P < 0.0001). **(d)** Representative staining for B-cells (CD19) and T-cells (CD3) in thymus and spleen. Scale bar: 100 µm.

In line with previous publications, thymi were 2.3-fold (*P* < 0.0001) smaller in *Runx1*^*−/−*^ mice (Fig. 2a)^20,21^. Furthermore, whereas wildtype and *Runx1*^+/−^ thymi presented with a distinct cortex and medullary zone, loss of cortex and medulla structures was observed in *Runx1*^*−/−*^ *mice* (Fig. 2d).

In contrast, spleens were slightly enlarged in *Runx1*^*−/−*^ mice as compared to *Runx1*^+/−^ or wildtype mice due to expansion of the myeloid compartment^20^. Moreover, spleens of *Runx1*^*−/−*^ mice displayed atrophic follicular structures associated with almost complete loss of splenic white pulp (Fig. 2d). The spleens of *Runx1*^+/−^ mice were similar to the wildtype architecture but with additional areas of atrophic follicular structures and loss of splenic white pulp (Fig. 2d).

IHC for the detection of the B-cell marker CD19 revealed a marked reduction of B-cells in the spleens of *Runx1*^+/−^ mice (1.4-fold, *P* = 0.0018) which was more pronounced in *Runx1*^*−/−*^ (2.8-fold, *P* < 0.0001) as compared to wildtype mice (Fig. 2b) and in line with previous reports^21^. The *a priori* relatively low number of B-cells in thymi was not significantly altered by *Runx1* knockout (Fig. 2b). IHC for the T-cell marker CD3 revealed a lower frequency of T-cells in the spleens of *Runx1*^+/−^ mice (1.3-fold, *P* = 0.0019) and in *Runx1*^−/−^ mice (2.6-fold, *P* < 0.0001) compared to wildtype mice (Fig. 2c). Furthermore, a strong reduction of the number of thymic T-cells was observed in *Runx1*^−/−^ mice (4.8 fold, *P* < 0.0001) in comparison to *Runx1*^+/−^ and wildtype mice (Fig. 2c). It is noteworthy that the intensity of the CD3 staining was much lower in the T-cells of *Runx1*^−/−^ mice.

**Figure 3:**
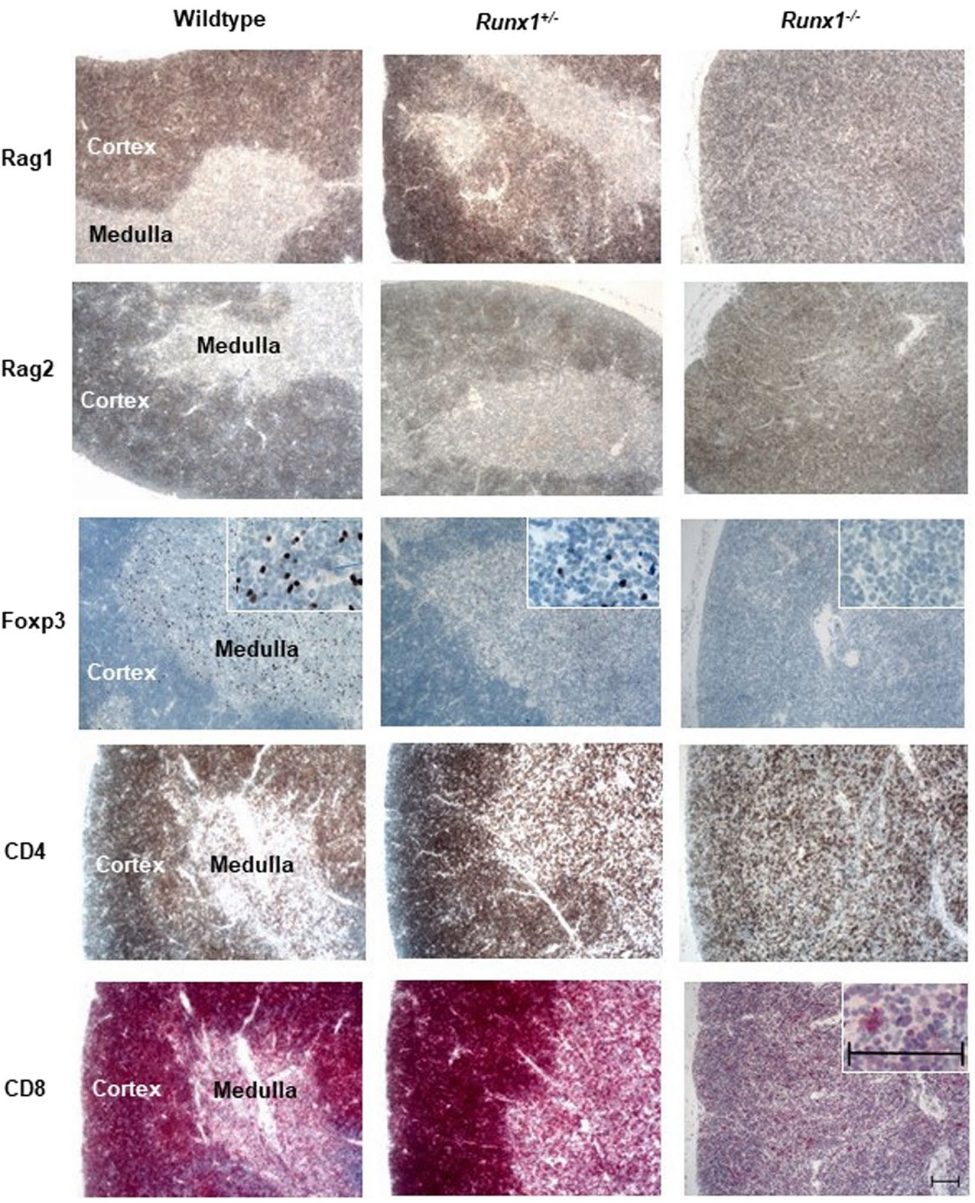
Representative immunostaining of Rag1, Rag2, Foxp3, CD4 and CD8 in thymi of wildtype, *Runx1*^+/−^ and *Runx1*^−/−^ mice. The insert (CD8 expression) demonstrates an almost exclusive CD8 expression in antigen-presenting cells but not in lymphocytes of *Runx1*^−/−^ thymi. The inserts (Foxp3 expression) show the decrease in Foxp3 positive cells in Runx1^+/−^ and Runx^−/−^ thymi compared to wildtype thymus. Scale bars: 100 µm.

The distribution of Rag1 and Rag2 positive cells in the thymi of wildtype and *Runx1*^+/−^ mice was restricted to the cortex and was absent in the medulla (Fig. 3), which is – for *Runx1* wildtype mice – in line with published data and the fact that TCR rearrangements take place in the cortex^19^. Rag proteins were equally strong expressed irrespective of Runx1 expression, however Rag-positive cells were scattered throughout the entire thymus of *Runx1*^−/−^ mice without association to a morphological structure, due to an absence of the medulla (Fig. 3).

T-cells undergo positive and negative selection in the thymic cortex and medulla, respectively^19^. In the medulla double positive CD4/CD8 T-cells further develop into CD4 or CD8 single positive T-cells^19^. If the T-cells of the medulla bind the antigen-MHC complex with high functional avidity, T-cells undergo apoptosis or become FOXP3 expressing regulatory T-cells to prevent autoimmune reactions^22^. Compared to wildtype mice, Foxp3 positive T-cells in the medulla were reduced by 1.4-fold (*P* < 0.02) in *Runx1*^+/−^ mice and almost absent in *Runx1*^−/−^ mice (Fig. 3, Supplementary Fig. S1).

RUNX1 is known to regulate various T-cell-specific genes such as CD4 and CD8 and interact with FOXP3^23,24^. Strikingly our IHC analysis revealed an almost complete absence of CD8 positive T-cells in thymi of *Runx1*^−/−^ mice whereas *Runx1*^+/−^ and wildtype mice displayed very similar number of CD8 T-cells (Fig. 3). CD4 positive T-cells were less strongly reduced in *Runx1*^−/−^ mice leading to a much higher CD4/CD8 ratio (Fig. 3).

In summary, the *Runx1* double knockout led to prominent thymic atrophy with a complete loss of the medulla. A massive reduction of the number of T-cells - most pronounced for single positive CD8 and Foxp3 regulatory T-cells - was observed in addition to the loss of splenic B lymphocytes. The *Runx1*^+/−^ mice showed a weak but significant reduction of B- and T-cells in the spleen.

### *Runx1* knockout affects the murine TCRβ repertoire and VDJ architecture

To test the hypothesis that RUNX1 is a recombinase cofactor for antigen receptor gene rearrangements and impacting TCRβ richness and VDJ architecture, we comprehensively analyzed the entire murine TCRβ repertoire of wildtype and *Runx1* knockout mice employing a quantitative next generation sequencing (NGS)-based analysis (TCRsafe™, Supplementary Methods, Supplementary Fig. S2). Our approach comprised the entire TCRβ complementarity determining region 3 (CDR3) and short proportions of the respective TCRβ V segments and J segments derived from the T-cells of thymi and spleens from *Runx1*^+/−^*, Runx1*^−/−^ and wildtype mice (N = 10 in each of the 6 groups; total number: 60 samples). A mean of 503,199 joint sequence read pairs (range 92,449 – 1,082,237) with TCRβ V and J segment matches were generated resulting in a mean of 10,257 (range 797–23,876) individual TCRβ gene rearrangements/clonotypes for each sample (clonotype frequency cut-off: 0.001%; Supplementary Table S1). The NCBI SRA accession number for the Fastq files of the 60 samples is PRJNA521529.

**Figure 4:**
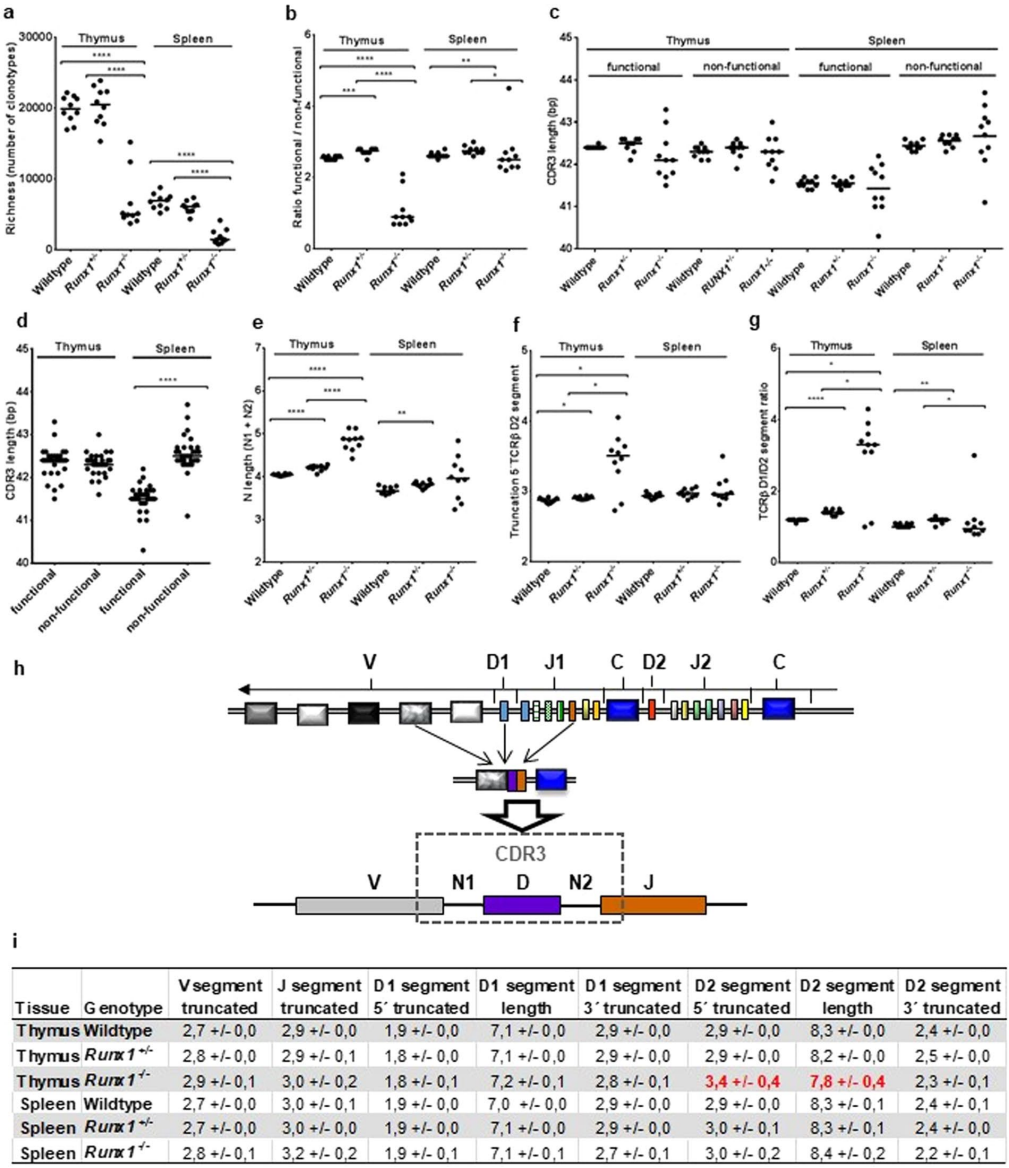
*Runx1* knockout affects the murine TCRβ repertoire and VDJ architecture. Dot plots depicting **(a)** TCRβ richness, **(b)** the ratio of functional versus non-functional TCRβ clonotypes, **(c)** the median CDR3 length in thymus and spleen being independent of the *Runx1* knockout status, **(d)** CDR3 length in functional and non-functional TCRβ clonotypes, **(e)** N length (N1 + N2), **(f)** 5′ truncation of the TCRβ D2 segment, (**g)** the TCRβ D1/D2 segment ratio. **(h)** Overview of the TCRβ locus and a schematic representation of the TCRβ rearrangement. **(i)** Table of TCRβ VDJ parameters with a more truncated 5′ D2 segment and a shorter D2 segment length in thymi of *Runx1*^−/−^ mice compared to *Runx1*^*+/*−^ and wildtype mice marked in red. Mean values and standard deviations were calculated from the 10 mean values of the 10 samples in each of the 6 groups (Supplementary Table S1). In all dot plots the median value is indicated (*P < 0.02, **P < 0.01, ***P < 0.001, ****P < 0.0001). Of note, the two outliers in the thymus sample of the dot plots a, b, f and g represented *Runx1*^−/−^ mice with a morphological phenotype in between the typical phenotype of *Runx1*^−/−^ and *Runx1*^+/−^ mice.

TCRβ richness was reduced 4.0-fold (*P* < 0.0001) in thymi and 4.4-fold (*P* < 0.0001) in the spleens of *Runx1*^−/−^ in comparison to *Runx1*^+/−^ and wildtype mice (Fig. 4a). In addition, there was a 2.9-fold (*P* < 0.0001) lower ratio of functional to non-functional thymic TCRβ rearrangements in *Runx1*^−/−^ mice compared to *Runx1*^+/−^ and wildtype mice leading to a proportion of approximately 50% non-functional TCRβ rearrangements in *Runx1* double knockout mice (Fig. 4b). This high rate of non-functional TCRβ rearrangements was only observed in thymi but not in the spleens of *Runx1*^−/−^ mice demonstrating that complete TCRβ VDJ rearrangements can be produced in the absence of RUNX1. However, the accumulation of non-functional TCRβ VDJ rearrangements in thymi of *Runx1*^−/−^ mice indicate that RUNX1 is important for the generation of proper recombination (recombinase activity). In addition, absence of RUNX1 also affects transcription factor activity which might additionally contribute to a disturbed T-cell selection process.

To elucidate the role of RUNX1 in the selection process of functional T-cells we next analyzed the TCRβ CDR3 length in the functional and non-functional TCRβ clonotypes of thymus and spleen. T-cells with non-functional TCRβ rearrangements are not selected by TCR MHC interaction in the thymus, whereas functional T-cells have the chance to be positively selected. Positive selection is described to result in a shorter CDR3 length^25,26^. In our mouse model, the median TCRβ CDR3 length was not significantly affected by the *Runx1* knockout status within the four groups of (i) thymic functional (ii) thymic non-functional (iii) splenic functional or (iv) splenic non-functional clonotypes (Fig. 4c). Interestingly, the variability of the CDR3 length increases from wildtype over *Runx1*^*+/*−^ and *Runx1*^−/−^ mice in thymus and spleen (Fig. 4c). This indicates a role of RUNX1 in controlled and optimized TCRβ CDR3 length as well as an involvement in the generation of a proper CDR3 repertoire. Of note, significantly shorter (*P* < 0.0001) TCRβ CDR3 lengths were only observed in functional TCRβ rearrangements in the spleen, regardless of the *Runx1* knockout status (Fig. 4c,d).

Next we analyzed in depth the TCRβ VDJ architecture (Fig. 4h). To this end we determined in each of the 6 subgroups the mean length of TCRβ N nucleotides, the TCRβ D1 and D2 segment length, as well as the V, J, and D segment truncation occurring during the recombination process (Supplementary Table S1). We observed 1.2-fold (*P* < 0.0001) longer N lengths in the thymi of *Runx1* knockout mice (Fig. 4e). In contrast there was 1.2-fold (*P* < 0.02) more extensive truncation at the 5′ end of the TCRβ D2 segment, leading to shorter TCRβ D2 segment lengths which was not observed for 5′ TCRβ D1 (Fig. 4f,i). There was also more marked truncation of V (thymus *P* < 0.0003, spleen *P* < 0.001) and J segments (thymus *P* < 0.47, spleen *P* < 0.0005) in Runx1^−/−^ mice compared to wildtype mice (Fig. 4i, Supplementary Table S1). The number of T-cells in the thymus carrying TCRβ D2 segments was reduced by 2.5-fold (*P* < 0.02). (Fig. 4g).

Taken together, we found that Runx1 loss led to an overall reduction of the TCRβ repertoire and to an altered CDR3 architecture due to an increase of V, 5′ D2 and J segment nucleotide excisions and N nucleotide additions. An increased variability in CDR3 length was observed for both, functional and non-functional, TCRβ clonotypes indicating that RUNX1 directly affects VDJ rearrangement. A more pronounced truncation at the 5′ end of the TCRβ D2 segments correlated with lower TCRβ D2 segment usage and therefore a shift towards a higher thymic TCRβ D1/D2 segment ratio (Fig. 4). The altered TCRβ D1/D2 segment ratio and VDJ architecture is in harmony with the role of RUNX1 as a recombinase cofactor.

### Binding of RUNX1 to evolutionary conserved RUNX1 heptamer motifs at the initiation site of TCRβ rearrangements

**Figure 5:**
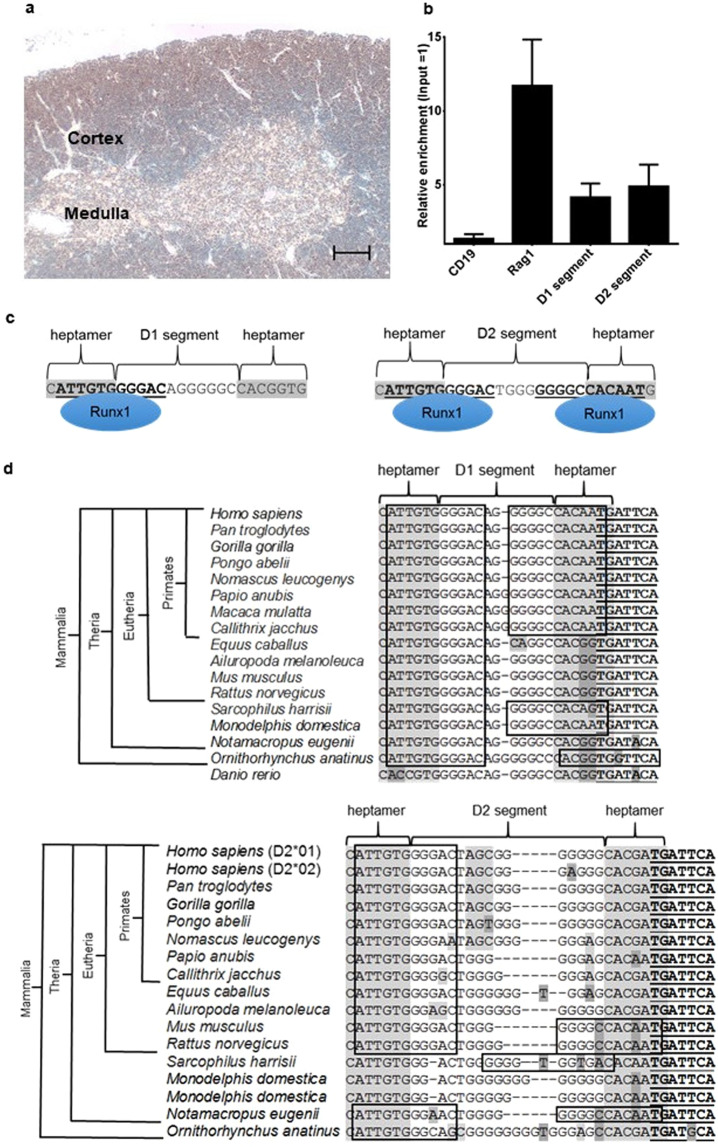
Binding of RUNX1 to evolutionary conserved RUNX1 heptamer motifs at the initiation site of TCRβ rearrangements. (**a**) Runx1 expression in the lymphocytes of the outer cortex and the medulla (IHC; Scale bar: 100 µm). (**b**) Runx1 ChIP analyzed by real-time DNA PCR. The *CD19* promoter was used as negative control and the *Rag1* core promoter, which harbors two adjacent Runx1 binding sites, as a positive control. ChIP enrichment was calculated as fold change relative to input DNA with error bars representing the standard deviation of three biological replicates. Input and the y-axis were set to 1. Runx1 binding was detected for the *Rag1* core promoter and the TCRβ D1 and D2 segments. (**c**) Model of Runx1 binding to the Runx1 heptamer motifs of the murine TCRβ D1 and D2 segments. **(d)** Alignment of TCRβ D1 and D2 segments and their adjacent RSS heptamers show that RUNX1 binding sites (indicated by squares) are highly conserved at the 5′ ends of D1 and D2 segments in Mammalia and Eutheria, respectively. At the 3′ borders of TCRβ D1 and D2 segments, RUNX1 heptamer motifs were found in approximately 45% of the analyzed mammalian species. In zebrafish (*Danio rerio*) used as outgroup only the TCRβ D1 segment and no D2 segment is present. Previously reported C-FOS binding motifs at the 3′ ends of the TCRβ D1 and D2 segments are bold and underlined^28^.

Runx1 expression was detected by immunostaining in the outer thymic cortex where TCRβ rearrangement is initiated (Fig. 5a)^27^. To further assess the role of Runx1 in the skewed TCRβ D1/D2 segment ratio in Runx1^−/−^ mice, we analyzed by chromatin immunoprecipitation (ChIP) the capability of Runx1 to directly bind the TCRβ D1 and D2 segments which have been reported to represent the initiation sites of TCRβ rearrangements^28^. Runx1 ChIP enrichment was detectable for TCRβ D1 and D2 Runx1 heptamer sequences as well as the *Rag1* core promoter (positive control) whereas no binding was found for the *CD19* promoter (negative control) (Fig. 5b). The binding of Runx1 to the two TCRβ D segments is graphically depicted in Fig. 5c.

By further comparison with published database sequences (www.ensembl.org), we found the RUNX1 heptamer motifs at the 5′ borders of TCRβ D1 and D2 segments to be highly conserved in Mammalia and Eutheria, respectively (Fig. 5d). The zebrafish (*Danio rerio*) genome, used for comparison as a more distantly related outgroup, harbored only a TCRβ D1 segment with a CACCGTG heptamer sequence with no overlapping RUNX1 sites. Compared to the highly conserved 5′ TCRβ RUNX1 heptamer motifs, the 3′ TCRβ D1 and D2 segments showed more variability and convergent evolution of RUNX1 heptamer motifs as exemplified by the CACAATG heptamer motif overlapping with a RUNX1 binding site at the 3′ TCRβ D1 segment of primates and the murine and rat TCRβ D2 segments (Fig. 5d).

These results demonstrate an evolutionarily conserved overlap of RUNX1 binding motifs with the heptamer sequences of the TCRβ D1 and D2 segments and Runx1 binding to the murine TCRβ D1 and D2 Runx1 heptamer motifs.

### Enrichment of RUNX1 motifs overlapping with heptamers at the borders of recurrent *ETV6-RUNX1* ALL deletions

The data presented are in line with the function of RUNX1 as recombinase cofactor that plays a major role for the generation of physiological deletions in the course of TCRβ rearrangements. However, it remained to be elucidated if RUNX1 is also involved in the initiation of pathological off-target deletions. To this end, we investigated the frequency of RUNX1 motifs overlapping with either single heptamer sites or heptamers associated with RSS modules at the borders of recurrent *ETV6-RUNX1* ALL deletions in comparison to corresponding motifs from the whole genome. Heptamers within RSS modules had an adjacent nonamer motif separated by a spacer with 12 +/−1 bp or 23 +/−1 bp length. As whole genome background (i) 4,671,973 heptamer hits (+/−7 nucleotides) and (ii) 167,320 heptamer hits (+/−7 nucleotides) within RSS modules were used (Supplementary Table S2).

We reanalyzed data of 56 published recurrent deletions subdivided into 14 clusters with 2–6 close located deletions previously identified in 51 cases of *ETV6-RUNX1* ALL^14^. It was shown that RSS and heptamer motifs without nonamer sequences are enriched at these deletion ends as compared to control sequences^14^.

In line with a role of RUNX1 as a recombinase cofactor, we found that heptamer motifs and their adjacent regions are enriched for overlapping RUNX1 binding sites at the deletion borders as compared to corresponding heptamer and RSS backgrounds taken from the whole genome. Our analysis for RUNX1 motifs revealed that in *ETV6-RUNX1* ALL the sequences at the deletion borders display a 2.4-fold overrepresentation (Z-score 5.9) in 65 heptamer hits and 3.7-fold overrepresentation (Z-score 5.0) within 15 heptamer hits within RSS modules. A Z-score > 2 is significant compared to normal background distribution, which can be assumed for sufficiently large data sets. This is the case for the dataset with all heptamer regions but not for the heptamer regions in RSS models. However, for the latter, the probability of finding the observed RUNX1 hits is *P* < 0.00004 based on a binomial distribution (Supplementary Table S2).

**Figure 6:**
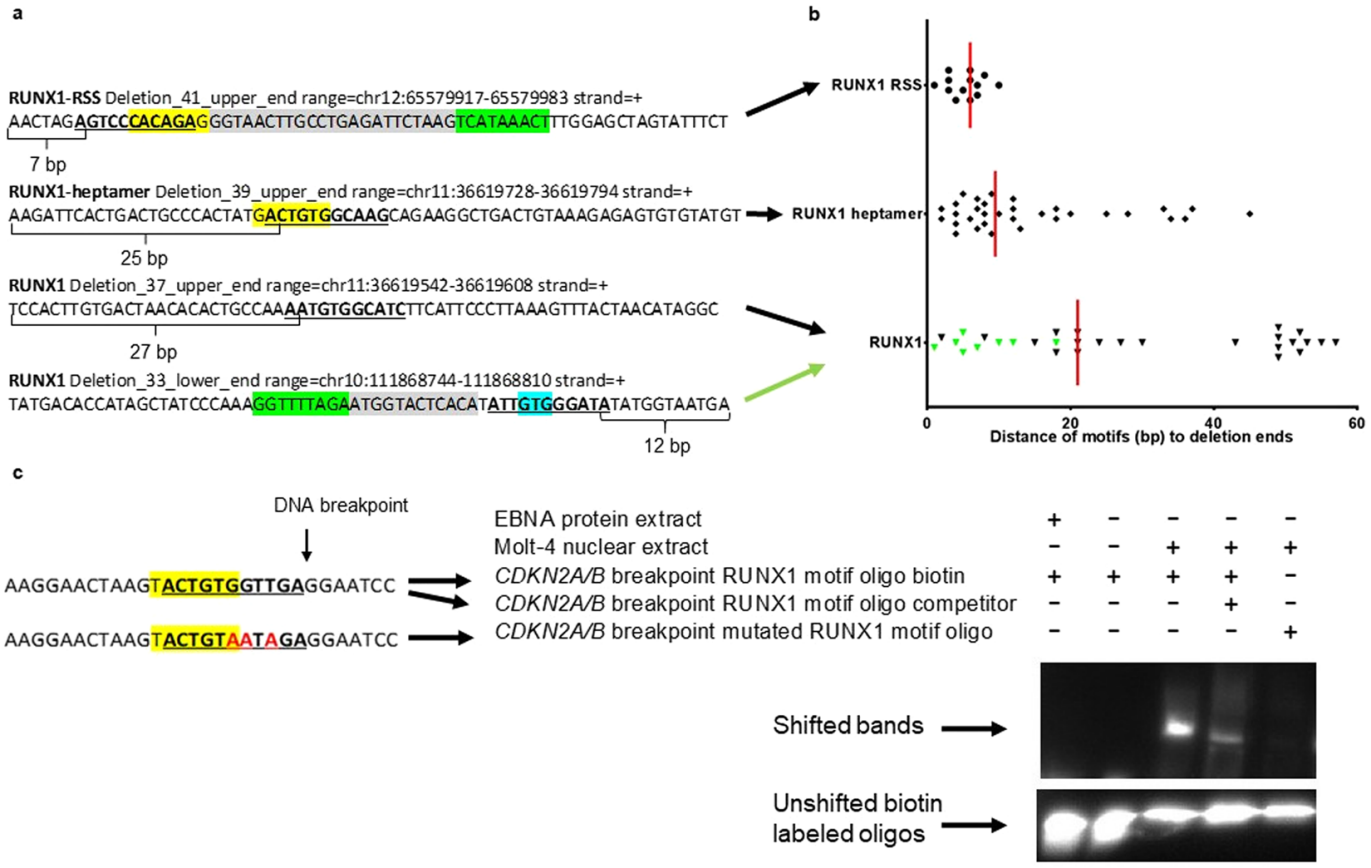
Spatial distribution of RUNX1 motifs at recurrent deletion breakpoints in *ETV6-RUNX1* ALL. **(a)** Mapping of RUNX1 (bold underlined), heptamer (yellow), spacer (grey), nonamer (green) and a putative partial GTG heptamer (blue) motifs onto 67 bp long deletion ends. The distance (bp) of the first nucleotide of the RUNX1 binding site to the 5′ or 3′ deletion end is indicated. **(b)** The distances (bp) of the RUNX1 motifs to the deletion ends are shown as dots for RUNX1 motifs (i) overlapping with a heptamer within a RSS module (RUNX1-RSS), (ii) overlapping with a heptamer without RSS module (RUNX1 heptamer) and (iii) not overlapping with a heptamer (RUNX1). Green dots represent RUNX1 motifs not overlapping a heptamer but with an adjacent nonamer in a spacer distance of 12 bp or 19–25 bp. Median values are indicated by a red line. **(c)** EMSA employing an oligonucleotide harboring a RUNX1 binding site (bold underlined) overlapping with a cryptic heptamer sequence at a recurrent breakpoint at the *CDKN2A/B* locus described for *ETV6-RUNX1* ALL and for CML^14,29^. As control, EBNA protein extracts (without RUNX1) show no shift of the biotin labeled oligonucleotide whereas a shift is seen with nuclear extracts of Molt-4 (expressing RUNX1). This shift is reduced by adding an unlabeled competitor and abolished by a mutated RUNX1 binding motif altered at 3 positions (indicated in red).

Next we analyzed the spatial distribution of RUNX1 heptamer hits at the border of the 56 recurrent deletions in *ETV6-RUNX1* ALL^14^. The distance of the identified RUNX1 binding sites from the deletion borders is depicted in Fig. 6 and describes RUNX1 motifs (i) overlapping with heptamer hits within RSS modules, (ii) overlapping with single heptamer hits and (iii) without heptamer overlap. RUNX1 heptamer hits within RSS modules were closest to the deletion borders, similar to heptamer hits without RSS modules (median 6 bp and 9.5 bp, respectively). Interestingly RUNX1 hits without overlapping heptamer motifs were more distant from the deletion borders (median 21 bp). Within the latter group, we discovered 8 RUNX1 binding sites (marked in green) with a putative conserved partial heptamer motif (GTG or CAC) being separated from an adjacent nonamer motif by a spacer of reasonable length (3 × 12 bp and 5 × 19–25 bp). These 8 RUNX1 motifs were significantly (*P* < 0.0001) closer to the deletion border (median 6 bp) as compared to RUNX1 motifs without an adjacent spacer-nonamer (median 36.5 bp). The genomic positions of heptamer, nonamer and RUNX1 motif hits at the borders of the 56 deletions are given in Supplementary Table S3.

### RUNX1 EMSA analysis of a recurrent *CDKN2A/B* breakpoint using Molt-4 nuclear extracts

To analyze the functional relevance of a RUNX1 binding motif at a recurrent, RAG-mediated *CDKN2A/B* breakpoint we performed an EMSA^14,29^. In the presence of Molt-4 nuclear extracts containing wildtype RUNX1, a shift in the mobility of the RUNX1 motif oligonucleotide was detectable. This mobility shift was strongly reduced by adding an unlabeled competitor and was completely abolished by using a mutated RUNX1 binding motif altered at 3 nucleotide positions (Fig. 6).

### Enrichment of RUNX1 heptamers within RSS modules in ChIP-Seq peaks of the ETV6-RUNX1 positive ALL cell line REH

To provide evidence for binding of the RUNX1-ETV6 protein to the RUNX1 binding motif in RSS we reanalyzed published 1,931 ChIP-Seq peaks derived from an ETV6-RUNX1 positive ALL cell line (REH)^30^. Although this ChIP-Seq was performed with a HA-tagged ETV6 bait protein, a significant enrichment of RUNX1 motifs was observed^30^. The authors interpreted this as evidence that the ETV6-RUNX1 fusion protein expressed in REH can interact with the ETV6-HA bait protein through its PNT domain, leading to ChIP-Seq data that comprises, in addition to ETV6 binding motifs, further ETV6-RUNX1 motifs^30^.

Using these 1,931 published ChIP-Seq peaks, we performed an enrichment analysis for overlapping RUNX1 motifs with heptamers associated with RSS modules and found 30 ChIP-Seq peaks associated with RSS modules. 15 of these 30 ChIP-Seq regions displayed RUNX1 motifs overlapping with the heptamer, compared to an expected number of 7.6 ± 2.7 SD, derived from the whole genome control. This represents a 2-fold enrichment (Z-Score 2.5, P = 0.01, Supplementary Table S2). Interestingly, two of the 15 RSS modules with overlapping RUNX1 heptamer sequences mapped to the TCRα V22 and Igλ V40 RSS, respectively.

### TCRβ rearrangements in *RUNX1* mutated and *RUNX1* wildtype human T-ALLs

To evaluate the impact of heterozygous *RUNX1* mutations on TCRβ rearrangements in T-ALL, 13 DNAs from *RUNX1* mutated T-ALLs (all heterozygous, 10 RHD domain, 3 TAD domain) were compared to 11 *RUNX1* wildtype T-ALLs, matched for an immature (IM) stage of maturation arrest, as defined by the absence of cytoplasmic or surface TCRβ protein expression^31^. The wildtype T-ALLs were selected to have at least incomplete clonal TCRβ rearrangements, to enable comparison of TCRβ D1 vs. D2 segment usage consisting of 5 T-ALLs with clonal TCRγ rearrangement (IMG) and TCRβ DJ rearrangement and 5 T-ALLs with a clonal TCRβ VDJ rearrangement (IMB) on at least one allele (Supplementary Table S4). The *RUNX1* mutated cases showed more diverse stages of maturation arrest, with 5 arrested prior to the onset of TCRβ rearrangement (3 IM0 with no clonal TCR rearrangement, 2 IMD with only TCRδ rearrangement), 3 IMG, 2 IMB, 2 pre-AB T-ALL (which expressed cytoplasmic but not surface TCRβ protein) and one which expressed a surface TCRγδ receptor (TCR-GD+). With regard to TCRβ rearrangements, the 13 *RUNX1* mutated T-ALLs demonstrated 12 clonal TCRβ rearrangements in 8 patients, including 9 TCRβ DJ and 3 TCRβ VDJ rearrangements. As expected, the latter were found in the T-ALLs with a more mature stage (Supplementary Table S4). Ten of the 12 (83%) rearrangements used the TCRβ D1 segment. All 11 *RUNX1* wildtype T-ALLs demonstrated at least one clonal TCRβ rearrangement by NGS (in the absence of expression of TCRβ protein), including 11 TCRβ DJ and 7 VDJ rearrangements. Of these 18 rearrangements, 11 (61%) used the TCRβ D1 segment, which was not significantly different from the TCRβ D1/D2 segment usage in *RUNX1* mutated T-ALLs (*P* = 0.25).

Regarding TCRβ J segment usage, *RUNX1* mutated cases used the TCRβ J1 cluster in 6/12 (50%) cases and *RUNX1* wildtype in 8/18 (44%) which was not significantly different (*P* = 1.0). There were too few clonal rearrangements to consider TCRβ CDR3 length or extent of 5′/3′ D segment truncations.

Taken together, the proportion of *RUNX1* mutated T-ALLs which undergo TCRβ rearrangement is lower (8/13; 62%) compared to T-ALL in general where over 80% are rearranged, as previously reported^31^. No significant difference could be detected for the TCRβ D1/D2 or J1/J2 segment usage in *RUNX1* mutated compared to *RUNX1* wildtype human T-ALLs.

## Discussion

Generation of functional TCRs requires the recombination of V, (D) and J TCR gene segments with excision of the intervening DNA sequences and the addition of random nucleotides at the V(D)J junction^16^. The excision of these DNA regions can be regarded as “physiological deletions”. This deletion process needs to be tightly controlled since misguided activity of the recombinase machinery might disturb the composition of the T-cell repertoire or – if directed against other genomic regions – many biological functions including cell proliferation and differentiation. At the core of the recombination machinery are the RAG proteins^15^. The *RAG* gene locus, comprising *RAG1* and *RAG2*, has been reported to be coordinately regulated by RUNX proteins and we confirm here Runx1 binding to the *Rag1* core promoter^32,33^. The transcription factor RUNX1 is also involved in the regulation of T-cell-specific genes such as CD4 and CD8^23,34^. Only recently an additional function for RUNX1 as a recombinase cofactor has been reported. By interaction with RAG1, RUNX1 leads to enhanced deposition of RAG1 to the human TCRδ D2 segment^18^. However, it remained unknown if RUNX1 also acts as a recombinase cofactor for TCRβ gene rearrangements or if this activity can be usurped to initiate non-physiological deletions.

To investigate the role of RUNX1 for TCRβ rearrangements, we employed a *Runx1* knockout mouse model combined with a deep sequence analysis of TCRβ gene rearrangements. In this mouse model an in-frame deletion of exon 5 of the Runx1 DNA-binding Runt domain was achieved in hematopoietic stem cells by Cre expression under the vav regulatory elements^20,21^. We demonstrated that in wildtype mice Runx1 is expressed in the sub-capsular region of the outer thymic cortex where TCRβ rearrangements are initiated and that the complete loss of Runx1 led to a highly disturbed thymic architecture and 4.8-fold reduction of mature CD3 positive T-cells. Interestingly, heterozygous loss of *Runx1* had little or no impact for the thymic architecture or T-cell content. In contrast, *Runx1*^−/−^ mice demonstrated an approximately 4-fold reduced TCRβ richness indicating that the absence of Runx1 leads to less effective TCRβ gene formation. This T-cell lymphopenia in *Runx1* knockout mice is thus likely attributable to the absence of the recombinase cofactor activity of Runx1. Especially as it has previously been published, in a *Runx*^F/F^ *Lck* –cre mouse model, that upon *Runx1* knockout a block in T-cell development occurs at the transition of the CD4/CD8 double negative (DN) stage 3 to DN4, that depends on assembly and expression of functional TCRβ rearrangements^34^. However, a contribution of the Runx1 transcription factor activity for the reduced T-cell number cannot be ruled out.

In the case that RUNX1 acts as a recombinase cofactor, its binding to the initiation site of TCRβ rearrangements at the D segment regions can be regarded as a prerequisite to induce excision at the TCRβ locus^28^. Indeed, our ChIP analysis revealed that Runx1 binds to murine TCRβ D1 segments at the 3′ Runx1 heptamer motif and to TCRβ D2 segments matching with 5′ and 3′ Runx1 heptamer motifs. The RUNX1 heptamer motifs at the 5′ borders of TCRβ D1 and D2 segments are highly conserved in Mammalia and Eutheria, respectively. Notably the zebrafish genome used for comparison as a more distantly related outgroup harbored only a TCRβ D1 segment with heptamers without overlapping Runx1 sites. Consistent with our model, *Runx1* knockout in zebrafish, having no Runx1 binding site overlapping its TCRβ D segment heptamers, was reported to have no impact on T-cell numbers and TCRβ rearrangements^35^.

Within Mammalia the occurrence of RUNX1 heptamer motifs at the 3′ borders of TCRβ D1 and D2 segments differs between species. A RUNX1 heptamer motif can be found at the 3′ border of the murine TCRβ D2 segment but not at the 3′ border of the human TCRβ D2 segment. Of note, we detected convergent evolution of RUNX1 heptamer motifs at the 3′ borders of TCRβ D segments (e. g. overlapping of the “CACAATG” motif with a RUNX1-binding site at the 3′ TCRβ D1 segment of primates and the murine and rat TCRβ 3′ D2 segment). Such convergent evolution strongly indicates a similar selection pressure in different mammalian species for the evolution of RUNX1 heptamer motifs adjacent to the TCRβ D segments.

The strongest evidence that RUNX1 directly impacts TCRβ CDR3 formation by its recombinase cofactor activity was provided by the observation of a higher CDR3 length variability in *Runx1*^−/−^ mice compared to wildtype mice. Notably these alterations were also observed in non-functional TCRβ rearrangement which are not affected by the TCR-MHC selection process. Furthermore, the CDR3 proportion is altered in the thymus of *Runx1*^−/−^ mice by 1.2-fold longer N regions, a 1.2-fold more extensive truncation at the 5′ end of the TCRβ D2 segment and a 2.5-fold reduction of TCRβ D2 segment usage. The more marked reduction in TCRβ D2 segment usage may reflect the presence of two Runx1 binding motifs compared to only one in TCRβ D1.

In essence, our data reflects the important role of RUNX1 for controlled and proper generation of the TCRβ repertoire and CDR3 composition. Thus, a malfunction of RUNX1 causing impaired TCRβ rearrangement is most likely also responsible for the high rate of non-productive TCRβ rearrangements (50%) observed in the thymi of *Runx1*^−/−^ mice.

Interestingly, the composition of the rearranged TCRβ sequences was different in the thymi and spleens of *Runx1* knockout mice. Significantly shorter (*P* < 0.0001) TCRβ CDR3 lengths were found only in functional TCRβ rearrangements of the spleen regardless of the *Runx1* knockout status. This is in agreement with previous publications reporting longer CDR3 lengths in T-cells of the thymus as compared to post-thymic T-cells^25,26^. In murine models, Yassai and colleagues described this TCRβ CDR3 shortening as a molecular marker for positive selection in later stages of maturation of CD4 single positive thymocytes^25^. Therefore, the shortening of the functional TCRβ CDR3 length in spleen as compared to thymic T-cells indicates that a positive selection process takes place independent of the *Runx1* knockout status. Thus, the accumulation of non-functional TCRβ VDJ rearrangements in the thymus of *Runx1* knockout mice argues for an important role for RUNX1 in the TCRβ rearrangement process but not for the T-cell selection process.

In contrast to the severe impact of the complete loss of Runx1 in *Runx1*^−/−^ mice on the TCRβ architecture and T-cell repertoire, the TCRβ composition was essentially unaffected in *Runx1*^+/−^ mice indicating that the presence of one intact allele is sufficient to exert the functions of RUNX1. This observation is in line with our finding that T-ALL patients with heterozygous *RUNX1* mutations display no difference in TCRβ rearrangements as compared to matched immature T-ALL samples with no evidence of *RUNX1* mutation.

Based on our finding that Runx1 impacts the physiological deletion processes in *Runx1*^−/−^ mice it was tempting to speculate that RUNX1 may under certain conditions also be involved in aberrant deletions events. To test this hypothesis, we investigated ALL cases with *ETV6-RUNX1* rearrangements (t(12;21)) comprising approximately 20% of all childhood ALL^11,12^. In these patients the *ETV6* protein dimerization domain is fused to the DNA binding and transactivation region of *RUNX1*. *ETV6-RUNX1* ALL is characterized by a variety of additional most likely secondary chromosomal alterations which consist of recurrent and private deletions. These deletions predominantly occur in promoters and enhancers of genes involved in B-cell differentiation and it was shown that RSS and single heptamer motifs are enriched at the deletion sites^14^.

Our reanalysis of published data comprising 56 recurrent deletions derived from *ETV6-RUNX1* ALL cases demonstrated the presence of RUNX1 heptamer motifs in close vicinity to these deletions^14^. A 2.4-fold overrepresentation (Z-score 5.9, *P* < 0.0000001) of RUNX1 binding sites overlapping with heptamers without adjacent nonamer motif and a 3.7-fold overrepresentation (Z-score 5.0, *P* < 0.00004) of RUNX1 heptamer motifs within a RSS were found in *ETV6-RUNX1* B-ALL deletion borders as compared to RUNX1 motif overlap with single heptamers and RSS backgrounds taken from the entire genome. Although this is not a functional proof, the significant enrichment of RUNX1 heptamer motifs at the deletion borders in conjunction with our *Runx1*^−/−^ mouse TCRβ data clearly argues for an involvement of RUNX1 not only in physiological deletions but also for a major role for the generation of pathological deletions in *ETV6-RUNX1* ALL.

Binding to the RUNX1 motif is not only possible for the native RUNX1 protein but also for the ETV6-RUNX1 fusion protein, which encodes almost the entire RUNX1 protein including its DNA-binding domain^36,37^. Furthermore, our reanalysis of ChIP-Seq data derived from the *ETV6-RUNX1* positive cell line REH suggests that ETV6-RUNX1 can bind to RUNX1 heptamer motifs in RSS of TCR and IG V-segments, indicating a role for the ETV6-RUNX1 protein in the ongoing TCR and IG rearrangements observed in *ETV6-RUNX1* ALL^13,30^. In addition, our EMSA experiments with Molt-4 nuclear extracts provide evidence that the RUNX1 heptamer site at a recurrent, recombinase mediated, *CDKN2A/B* breakpoint described for ETV6-RUNX1 ALL is functionally relevant^14^. However, the data derived from our *Runx1* knockout model also demonstrates that RUNX1 is not strictly essential for the physiological deletion process since TCRβ rearrangements still occur at very low frequency despite *Runx1* knockout. This is consistent with our finding in ALL where - despite significant enrichment of RUNX1 heptamers at deletion borders - some deletion sites do not demonstrate RUNX1 heptamer motifs.

**Figure 7:**
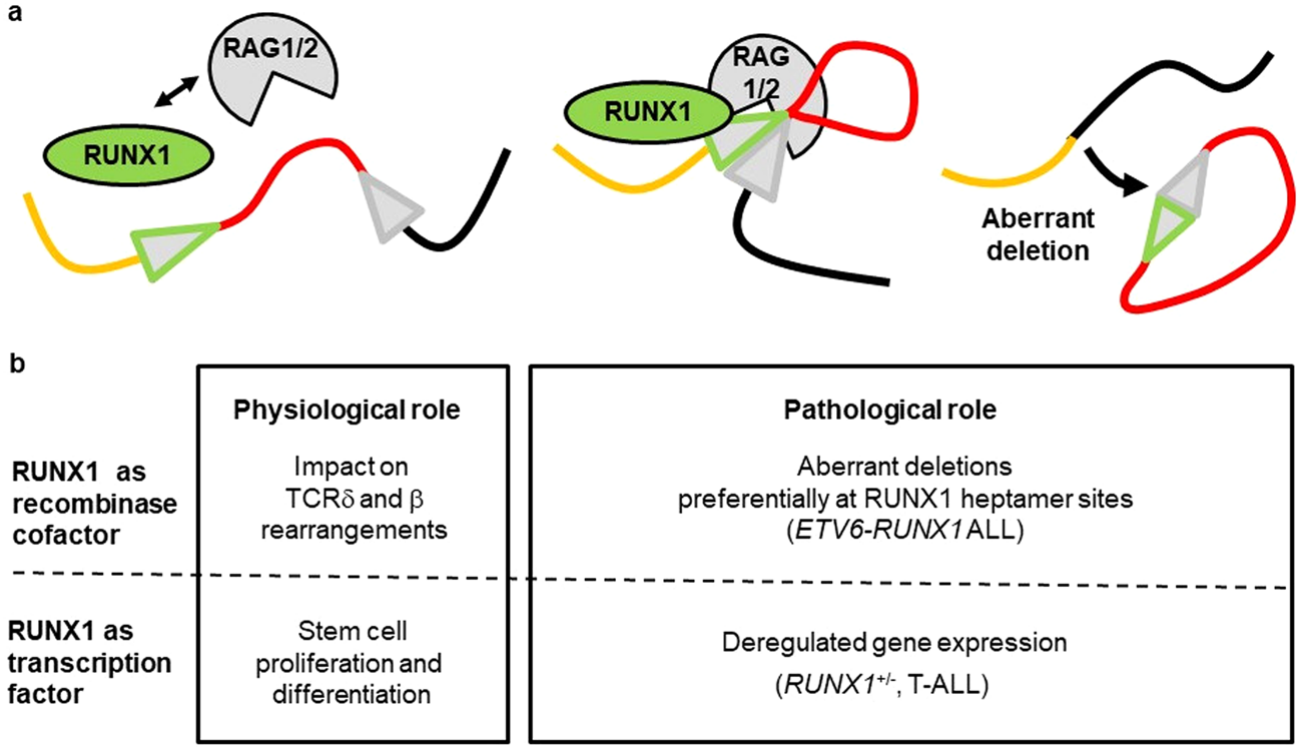
RUNX1 in physiological and pathological settings (**a**) Model for RUNX1 as recombinase cofactor during a pathological deletion process. A direct interaction of RUNX1 and RAG1 was previously shown^18^. In our model, RUNX1 can enhance the binding of RAG1/2 complexes at sites with RUNX1 heptamer motifs, leading to aberrant deletions (Green triangle: Overlapping RUNX1 and RAG binding site; grey triangle: RAG binding site). **(b)** Dual role of RUNX1 as transcription factor and recombinase cofactor under physiological and pathological conditions.

Therefore, we propose a model where RUNX1 (and/or ETV6-RUNX1) acts as an attractant for pathological deletion processes (Fig. 7). However, the model of cooperation between RAG1 and RUNX1 for exertion of pathological deletions remains unclear and deserves further exploration. According to the “nonamer first” model RAG1 interacts primarily with the nonamer sequence^38^. In this case the RUNX1 binding site overlapping with the heptamer is only separated by the RSS spacers of 12 or 23 bp length. Cieselak *et al*. already showed that RUNX1 and RAG1 can directly interact in the lymphoblastic cell line Molt-4 making it likely that RUNX1 can enhance RAG1/2 binding and initiation of deletions^39^.

Taken together we propose a role of RUNX1 as recombinase cofactor in addition to its well-established function as transcription factor (Fig. 7). In our model, RUNX1 acts as a recombinase cofactor at early stages of lymphoid differentiation when the formation of functional TCR gene rearrangements is a crucial step for T-cell survival and development^16,17^. Moreover, RUNX1 may also act as a recombinase cofactor decisive for recurrent pathological deletions in *ETV6-RUNX1* ALL by enhancing RAG binding at *RUNX1* heptamer binding sites. Thus, RUNX1 needs to be taken into consideration as a recombinase cofactor for a better understanding of molecular mechanisms leading to physiological (e.g. TCRβ, TCRδ) and aberrant (e.g. *ETV6-RUNX1* ALL) deletions.

## Material and Methods

### Experimental animals

We performed TCRβ repertoire (NGS) and immunohistochemical analyses (IHC) on spleen and thymus tissues of littermates from B6.*Runx1*^*fl/+*^-Tg(*vav*-Cre) X B6.*Runx1*^*fl/+*^ crosses^21^. (Unique identifier MGI:3798051, available upon request, C.S.). Only offspring carrying the Tg(vav-Cre) allele were used for analysis. The Cre recombinase is expressed under the *Vav* promoter, which is first expressed in the hematopoietic stem cells within the fetal liver, which give rise to all definitive hematopietic cells^21,40^. Cre expression inactivates the floxed *Runx1*^*fl*^ allele by deletion of exon5, encoding the essential Runt DNA-binding domain. Consequently, all definitive (adult) hematopoietic cells in these mice will have a *Runx1*^−/−^. *Runx1*^+/−^, or wildtype Runx1 genotype.

All animal strains were maintained at the animal facility of the Heinrich-Pette-Institute. All animal studies were approved by the Hamburg commission for animal experiments (Nr. 89/13) and the LaGeS0 (T0114/05). All experiments were performed in accordance with relevant guidelines and regulations. In total 30 mice (10 wildtype, 10 *Runx1*^+/−^, 10 *Runx1*^−/−^) with the age of 7 weeks, were analyzed. In addition, the thymi of 6–7 weeks old wildtype mice were used for Runx1 ChIP-analysis.

### Immunohistochemistry

Immunostaining of thymus and spleen tissue was performed on consecutive sections, using the BondMax™ device (Leica Biosystems) employing the manufacturer´s protocol and reagents. For primary antibodies, dilutions of 1:500 were used for CD4 (rabbit monoclonal, ab183685, Abcam) and CD19 (rat monoclonal, 60MP31, Thermo Fisher) antibodies. A dilution of 1:200 was used for CD8 (rat monoclonal, DIA-808, Dianova), RAG1 (rabbit monoclonal, DCABH-5798, Creative Diagnostics), RAG2 (rabbit polyclonal, LS-C408005, Biozol) and CD3 (rabbit polyclonal, ab5690, Abcam) antibodies. The FOXP3 rabbit monoclonal antibody (D6OR, Cell Signaling) was diluted 1:100. For antigen retrieval (20 minutes) the Bond-ER2 antigen retrieval solution (Leica Biosystems) was employed for all antibodies. The visualization of rabbit primary antibodies was performed by using the BOND Refine Detection System (DS9800, Leica Biosystems) including an anti-rabbit-HRP antibody and DAB/Hämalaun. The detection of rat primary antibodies was realized by using a rabbit anti-rat-bridge (E0468, Dako) followed by the BOND Refine Detection System as described above. The slides were evaluated independently by two pathologists. For quantification of CD19, CD3 and FOXP3 the mean of the two analyses was visualized as dot plots employing GraphPad PRISM V.6 software (GraphPad Software). Non-parametric analysis between data groups were performed by two-tailed Mann-Whitney U tests. The medians were used to calculate fold changes. If two groups were compared to another group (e.g. wildtype and *Runx1*^+/−^compared to *Runx1*^−/−^) the mean of the first two medians (e.g. wildtype and *Runx1*^*+/*−^) was employed to calculate the fold changes with the median of the other group (e.g. *Runx1*^−/−^).

### Analysis how *Runx1* knockout affects the murine TCRβ repertoire and VDJ architecture

Genomic DNA was extracted from formalin-fixed paraffin-embedded thymus and spleen tissue samples employing the Maxwell RSC FFPA DNA kit (Promega). A TCRsafe™ analysis was established to quantitatively analyze the murine TCRβ repertoire (Supplementary Methods and Results, Supplementary Fig. S2, Supplementary Table S5). To generate the NGS libraries a cross-contamination protected two-step PCR was utilized as described previously^41^. The primary PCR amplification (PA) were set up with AmpliTaq-Gold (Thermo Fischer Scientific), TCRsafe™ PA buffer (HS Diagnomics), 3.2 µM Primermix and 400 ng DNA template in a final volume of 50 µL. The cycling conditions were: 95 °C 12 min, 27 cycles of 95 °C 40 s, 64 °C 1 min, and 72 °C 1 min, with final extension at 72 °C for 7 min. In the second amplification (SA), adaptor sequences required for NGS were introduced, with Phusion High-Fidelity DNA Polymerase (Thermo Fischer Scientific), TCRsafe™ SA buffer (HS Diagnomics), with forward and reverse primers (1 µM each), in a final volume of 25 µL, using a 1:100 dilution of the corresponding primary PCR products as template. The cycling conditions were as follows: 98 °C 2 min, 5 cycles of 98 °C 20 s, 58 °C 30 s, and 72 °C 30 s, 5 cycles of 98 °C 20 s, 63 °C 30 s, and 72 °C 30 s, 13 cycles of 98 °C 20 s and 72 °C 30 s with final extension at 72 °C for 5 min. Spin column-purified PCR products were sequenced with MiSeq (Illumina) in paired-end mode (2 × 150 bp). For multiplexing standard TruSeq barcodes and MiSeq sequencing default conditions were employed allowing one mismatch in the barcode.

Bioinformatics analysis was performed employing the proprietary TCR integrate software (HS Diagnomics). In brief, read pairs delivered by the Illumina sequencer in paired-end mode were joined using the phred-like quality values per base to deliver a proper consensus sequence per single read pair. The joined reads were clustered and classified with respect to the TCRβ V and J segments as previously described^41^. To reduce the effect of PCR errors, low abundance clonotypes that differed only by 1 bp from another clonotype were merged with the most abundant related clonotype if the latter was at least 20-fold more frequent. Furthermore, a clonotype frequency of 0.001% was employed as cut-off for all analyses. The TCRβ CDR3 proportion was defined starting from the conserved 5´ cysteine in the TCRβ V segment and ending at the conserved 3′ phenylalanine in the TCRβ J segment. To define the presence of a D segment a minimum length of 5 nucleotides was used.

Noteworthy the TCRβ analysis was focused on all functional TCRβ alleles present in the C57Bl/6 mouse line used in this study. The TCRβ reference sequences for the C57Bl/6 mouse line were derived using the IMGT data base (http://www.imgt.org/IMGTrepertoire) in combination with ensemble Blat searches for the C57Bl/6 mouse reference genome (https://www.ensembl.org).

To analyze the impact of *Runx1* knockout on the TCRβ rearrangements we performed the following comparative analyses in the six groups of wildtype, *Runx1*^*+/*−^ and *Runx1*^−/−^ samples derived from thymus and spleen (N = 60): The total number of clonotypes (richness) was calculated and for each TCRβ rearrangements the truncation of V, J and D segments and the length of N1 and N2 and the CDR3 region was determined. The ratio of functional/non-functional TCRβ rearrangements and the ratio of TCRβ D1/D2-segment usage was assessed.

Results were visualized as dot plots employing GraphPad PRISM V.6 software. Non-parametric analysis between data groups were performed by two-tailed Mann-Whitney U tests. The medians were used to calculate fold changes. If two groups were compared to on other group (e.g. wildtype and *Runx1*^+/−^ compared to Runx1^−/−^) the mean of the two medians of the first group was employed to calculate the fold change between the two groups.

### ChIP analyses for the detection of Runx1 DNA binding to TCRβ D1 and D2 segments

To better understand the effects of Runx1 on the murine TCRβ repertoire we performed ChiP employing the polyclonal rabbit RUNX1 antibody (ab23980, Abcam). Fresh-frozen mouse thymus was thawed on ice and cut into small pieces with a scalpel. The tissue parts were resuspended in 1.5 ml of ice-cold PBS (containing protease inhibitor) and incubated after addition of 150 μl of 11% formaldehyde solution at room temperature for 10 minutes. Quenching was achieved by adding 86.8 μl of 2.5 M glycine. The tissue was homogenized by a douncer followed by filtration through a 40 μm cell strainer. The filtrate was washed with PBS and cell amount was determined. Finally, approximately 2 × 10^7^ cells were frozen down in aliquots until the ChIP experiment was carried out. 2 µg antibody were used for an individual ChIP experiment. In addition, input DNA without ChIP enrichment was used for comparison. ChIP with three biological replicates were carried out according to the protocol developed by R.A. Young and colleagues with minor modifications^42,43^. In brief, isolated DNA was first incubated overnight at 4 °C with the RUNX1 antibody. Subsequently, the DNA/antibody mixture was incubated for further 3 hours at 4 °C with the protein G beads. Finally the DNA/antibody/bead mixture was washed 4 times with 10 mM Hepes, 500 mM LiCl, 1 mM EDTA, 1% Igepal CA-630, 0,7% sodium lauryl sulfate, pH 7.6 and DNA purification was performed using a phenol:chloroform:isoamyl alcohol precipitation.

ChIP enrichment of DNA fragments was determined by real-time DNA-PCR with primers covering the Runx1 binding sites in the TCRβ D1 and D2 heptamer sequences as well as the *Rag1* core promoter. The B-cell specific *CD19* promoter and a 3′ region of the *Prame* gene was used for comparison. Real-time DNA-PCR was performed with PowerUp SYBR Green Master Mix (Biosystems) on a Step One Plus Real-Time PCR Systems (Thermo Fisher Scientific) using the PCR parameters recommended by the manufacturer.

Relative quantification of real-time DNA-PCR results was calculated using the comparative ΔΔCT method^44^. All primers employed were tested to display an amplification efficiency of approximately 100% (+/−10%). Primer sequences are available from Supplementary Table S6.

### Evolutionary analysis of RUNX1 binding sites in TCRβ D1 and D2 segments

To analyze if the RUNX1 binding sites adjacent to the murine TCRβ D1 and D2 segments are evolutionary conserved, we performed BLASTN searches (www.ensembl.org) using the murine TCRβ D1 and D2 segment with their adjacent RSS (including the spacers and nonamer motifs) as query sequences. As modification, the D2 segment with adjacent RSS from *Notamacropus eugenii* was used to detect the D2 segment in the genomes of *Sarcophilus harrisii*, *Monodelphis domestica* and *Ornithorhynchus anatinus*. The TCRβ D1 and D2 sequences were aligned manually. We employed Genomatix Software Suite v3.10 (Precigen Bioinformatics Germany, formerly known as Genomatix) to determine RUNX1 binding sites (RUNX1 matrices V$AML1.01 and V$AML1.02 with a similarity threshold of 0.8) and Consite (http://consite.genereg.net/cgi-bin/consite) employing default conditions to predict C-FOS binding sites.

### Enrichment of RUNX1 motifs overlapping with heptamers at the borders of recurrent *ETV6-RUNX1* ALL deletions

To address the question if a RUNX1 heptamer fingerprint marks the borders of recurrent *ETV6-RUNX1* ALL deletions we reanalyzed published data by Papaemmanuil and colleagues^14^. They described 56 recurrent deletions subdivided into 14 clusters, which were identified based on 51 cases of *ETV6-RUNX1* ALL. They proved that RSS and heptamer motifs without nonamer sequences are enriched at this deletion ends compared to control sequences^14^. To determine if RUNX1 motifs overlapping with single heptamers or heptamers within a RSS are significantly enriched at the deletion borders compared to corresponding backgrounds taken from the whole genome, we employed the Genomatix Software Suite v3.10 (Precigen Bioinformatics Germany)^45,46^.

To this end, Genomatix MatDefine was used to generate two heptamer positional weight matrices from corresponding sequences in the TCRβ, TCRδ and IGH 3′ RSS of the D segments and the 5′ RSS of J segments^45^. Likewise, two nonamer positional weight matrices were created (Supplementary Table S7). Specifically, the corresponding unique sequences were used as input for the matrix generation. The matrix sequence logos are shown in Supplementary Fig. S3. Genomatix FastM was employed to create eight RSS models based on the heptamer and nonamer matrices (Supplementary Table S8)^46^. The reasoning to use the heptamer and nonamer motifs relevant for D to J segment rearrangements was that DJ rearrangements occur before V segment rearrangements and thus the 3′ D segment and 5′ J segment RSS are most relevant for the initiation of the physiological deletion process.

Next the whole genome and the recurrent *ETV6-RUNX1* ALL deletion ends were scanned for matches to the heptamer and RSS matrices using Genomatix MatInspector^45^. We analyzed 60 bp of the deletion ends and added 7 bp to the 5′ deletion ends and 7 bp to the 3′ deletion ends, resulting in 67 bp long deletion end regions. The reason to add 7 bp at the border of each deletion end was that a 4 nucleotide overlap between the heptamer and RUNX1 motif was demanded and the employed RUNX1 binding site had a length of 11 nucleotides. Therefore, in the analysis 7 nucleotides were also added to each side to each heptamer sequence match. If sequences overlapped to another extracted sequence by at least one nucleotide, the sequences were merged to avoid duplications. RUNX1 matches were defined by a matrix family consisting of the RUNX1 matrices V$AML1.01 and V$AML1.02 from Genomatix MatBase with a similarity threshold of 0.8.

The sequences extracted from the whole genome were used as background to calculate an overrepresentation of RUNX1 at the deletion borders of *ETV6-RUNX1* ALL overlapping the heptamers. This overrepresentation was calculated (i) for heptamer sequences within RSS module hits and (ii) for all heptamer hits. For defining module matches in the deletion borders and in the genomic background, Genomatix ModelInspector was used^46,47^.

### Enrichment of RUNX1 heptamers within RSS modules in ChIP-Seq peaks of the *ETV6-RUNX1* positive ALL cell line REH

With the same methods described in the previous paragraph, we reanalyzed 1,931 published ChIP-Seq peaks of the *ETV6-RUNX1* positive ALL cell-line REH^30^. We focused on the determination of RSS within the ChIP-Seq peaks, employing Genomatix ModelInspector to identify module matches and subsequently performed an enrichment analysis for RUNX1 motifs overlapping with heptamers within RSS modules^46,47^.

### RUNX1 EMSA analysis of a recurrent *CDKN2A/B* breakpoint with Molt-4 nuclear extracts

Biotin labeled forward and reverse oligonucleotides (30 bp length) were synthesized (Sigma-Aldrich) with the sequence of a published recurrent deletion border at the *CDKN2A/B* locus^14,29^. These oligonucleotides harbored the RUNX1 heptamer binding site in the middle (Fig. 6). The two oligonucleotides were also synthesized as unlabeled competitors. As further control, a forward and reverse biotin labeled oligonucleotide with a mutated RUNX1 binding site (alteration at 3 nucleotide positions) was employed (Fig. 6). The corresponding forward and reverse oligonucleotides were annealed in a thermal cycler by a first step of 5 min at 95 °C followed by 70 cycles with a decrease of 1 °C each step and final cooling down to 4 °C.

Nuclear extracts were prepared from the Molt-4 T-ALL cell line employing NE-PER nuclear and cytoplasmic extraction reagents (Thermo Fisher) and stored at −80 °C after snap freezing with 10% glycerol in liquid nitrogen.

Binding reactions were performed using the Lightshift Chemiluminescent EMSA Kit (Thermo Fisher) according to the manufacturer’s instructions with the following specifications: 1X binding buffer, 2.5% glycerol, 5 mM MgCl_2_, 75 ng/mL Poly(dI-dC), 0.05% NP-40, 2.5 µg/µl BSA, 4 µg nuclear extracts and 10 fmol biotin-labeled probe in a total volume of 20 µl. For competition the unlabeled probe was added to the reaction mixture prior to addition of the labeled probes with an excess of 400-fold molar concentration. After binding reaction, the samples were gently mixed with 5 µl 5X loading buffer and loaded onto a 6% DNA-retardation gel (Invitrogen). Gel running was performed using 0,5X TBE buffer and 110 V. Blotting was realized by using a 0.45 µm Biodyne B nylon membranes (Thermo Fisher), a XCell SureLock Mini-Cell system (Invitrogen) and blotting time of approx. 45–60 minutes at 100 V with 0.5X TBE buffer. Subsequently, UV-crosslinking of the membrane was done at 120 mJ/cm2 for 45 sec prior to chemiluminescence detection using HRP substrate solution (Thermo Fisher) and FusionCapt Advance analysis Software (Fusion device, Vilber Lurmat GmbH).

### Analysis of TCRβ rearrangements in *RUNX1* mutated and *RUNX1* wildtype T-ALLs

T-ALL samples were obtained at diagnosis from protocol treated children and adults for oncotyping and minimal residual disease quantification^48,49^. The multicentric trials were registered at ClinicalTrials.gov (GRAALL-2003, NCT00222027; GRAALL-2005, NCT00327678) and approved by local and multicenter research ethical committees. All experiments were performed in accordance with relevant guidelines and regulations. TCRβ rearrangements and their CDR3 junctions were compared in 13 T-ALLs with mono-allelic, acquired *RUNX1* mutations and 11 T-ALLs with *RUNX1* wildtype status. The *RUNX1* mutation status for these cases was known due to a previous study^50^. TCR rearrangements were analyzed by Genescan analysis after EuroClonality multiplex PCR and by next generation amplicon sequencing of TCRβ DJ and VDJ rearrangements followed by Vidjil bioinformatics analysis^51–53^. Two-tailed Fisher exact tests were performed to compare the characteristics of the TCRβ rearrangements between the two groups.

## Supplementary information

Supplementary_Data.

Supplementary_Table_S1.

Supplementary_Table_S3.

Supplementary_Table_S4.

## Data Availability

Sequencing data supporting the findings of this study have been deposited in the NCBI SRA data base (www.ncbi.nlm.nih.gov/sra/, TCRβ mouse data, accession number: PRJNA521529 and TCRβ T-ALL data, accession number: PRJNA509233). All other relevant data are available as supplementary data or from the corresponding authors upon reasonable request.
