# Supplementary material for: Evidence for a role of RUNX1 as recombinase cofactor for TCRβ rearrangements and pathological deletions in ETV6-RUNX1 ALL
Source: Sci Rep. 2020 Jun 22;10:10024. doi: 10.1038/s41598-020-65744-0 (PMC7308335; doi:10.1038/s41598-020-65744-0)
Supplement: Supplementary file 1 — Supplementary_Data. [file 41598_2020_65744_MOESM1_ESM.docx]

**SUPPLEMENTARY DATA**

**Evidence for a role of RUNX1 as recombinase cofactor for TCRβ rearrangements and pathological deletions in ETV6-RUNX1 ALL.**

**Authors:** Seitz V^1,2, §^, Kleo K^1, §^, Dröge A^2^, Schaper S^2^, Elezkurtaj S^1^, Bedjaoui N^3^, Dimitrova L^1^, Sommerfeld A^1^, Berg E^1^, von der Wall E^1^, Müller U^4^, Joosten M^1^, Lenze D^1^, Heimesaat MM^5^, Baldus C^6^, Zinser C^7^, Cieslak A^3^, Macintyre E^3^, Stocking C^8^, Hennig S^2^, Hummel M^1*^

**Supplementary Methods**

**Establishment of a TCRsafe™ PCR to quantitatively analyze the murine TCRβ repertoire.** On the basis of the murine TCRβ annotations by the IMGT collaboration (www.imgt.org) we designed 21 forward primers and 15 reverse primers that anneal to all alleles of 23 known functional TCRβ V segments and 13 functional J segments respectively (Supplementary Table S5). Primers were calculated to be T_m_-matched and to yield similar product lengths for all TCRβ V–J-combinations with the intension to amplify all functional murine TCRβ rearrangements.

However, multiplex PCR can introduce significant amplification biases that alter the composition of sequence libraries prepared. To measure and overcome these technical constrains a synthetic TCRβ reference template was created representing the complexity of somatically rearranged immune receptor loci.

To this end, we used a synthetic murine immune receptor repertoire comprising individual TCRβ VDJ rearrangements (N = 84). The TCRβ V and J segment permutations are given in Supplementary Fig. S2. The reference template sequences consisted of an Illumina TruSeq NGS adaptor at both ends, a V segment proportion of 131 bp, a J segment proportion of 100 bp and an artificial NDN region of 30 bp, leading to a total length of 417 nucleotides. Overall each of the 21 V primers and 15 J primers matched 4 and at least 5 reference template sequences, respectively. The complementarity determining region 3 (CDR3) comprised from each TCRβ V segment and J segment 9 germline nucleotides 3´ of the conserved cysteine and 5´ of the conserved phenylalanine, respectively. Between the TCRβ V and J segment sequences the murine TCRβ D1 segment (12 bp length) was included flanked by 9 random nucleotides on each side. Each of the 84 sequences were synthesized by GeneArt and provided in a pEX-A2 vector. Following plasmid purification (Qiaprep Spin Miniprep Kit, Qiagen) the 84 plasmids were quantified by UV spectrometry before they were mixed in equal quantities.

To validate an equal composition of the cloned 84 different TCRβ VDJ reference sequences, digestion with *AarI* restriction enzyme and end repair (Klenow fragment, NEB) was performed prior to sequencing via TruSeq Illumina adaptors on a MiSeq (Illumina) in paired-end mode (2× 300 bp) according to the manufactures recommendations.

A two-step TCRsafe™ approach with PCR conditions as described in the method section of this manuscript and the reference template was employed to optimize a quantitative analysis of the murine TCRβ repertoire in 5 iterative steps. As starting point we used an equimolar primer mix to determine over- and under-amplification of TCRβ V or J segments by NGS. According to this data we adapted the concentrations of individual primer to minimize amplification bias. The optimized primer mixes were then used to amplify the reference template pool and determine the remaining amplification bias. If a TCRβ V or J segment was over- or under-amplified the respective primer concentration was decreased or increased, respectively, for the next optimization round. We continued this process until there was no further improvement of the uniformity.

**Synthetic TCRβ reference template analysis and TCRβ PCR optimization.** NGS analysis of the entire reference template without additional PCR amplification by using Illumina adaptors revealed that the 84 reference template fragments had an even distribution. (Blue bars in Supplementary Fig. S2).

As demonstrated in Supplementary Fig. S2 the PCR-based amplification bias of the murine TCRβ PCR was minimized by five rounds of optimization by adjustment of respective primer concentrations. While the amplification bias of the equimolar primer mix showed a range of > 900 between the most and less strongly amplified reference template fragment, the bias was reduced in the optimized primer mix to only 12 fold. Due to the impact of sequence compositions (e.g. GC frequency) on the amplification rate it was not possible to eliminate amplification differences completely.

**Supplementary Figures**

**
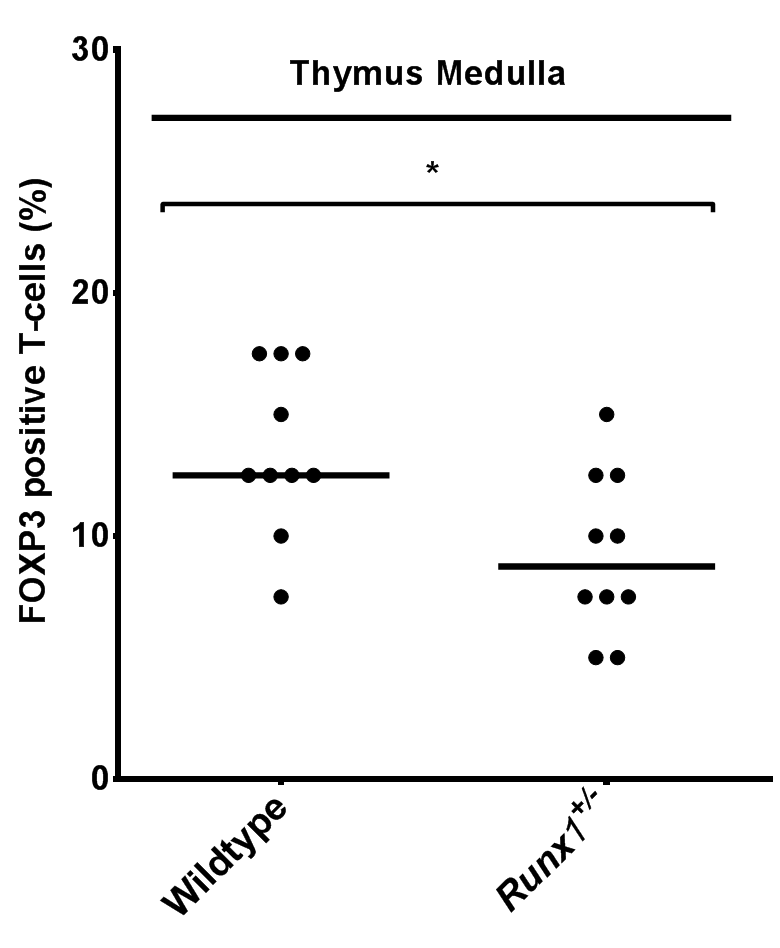
**

**Supplementary Figure S1**

**Impact of *Runx1* knockout on FOXP3 positive T-cells.** Compared to wildtype mice, Foxp3 positive T-cells in the thymic medulla were reduced in *Runx1^+/-^* mice 1.4-fold (*P** < 0.02).


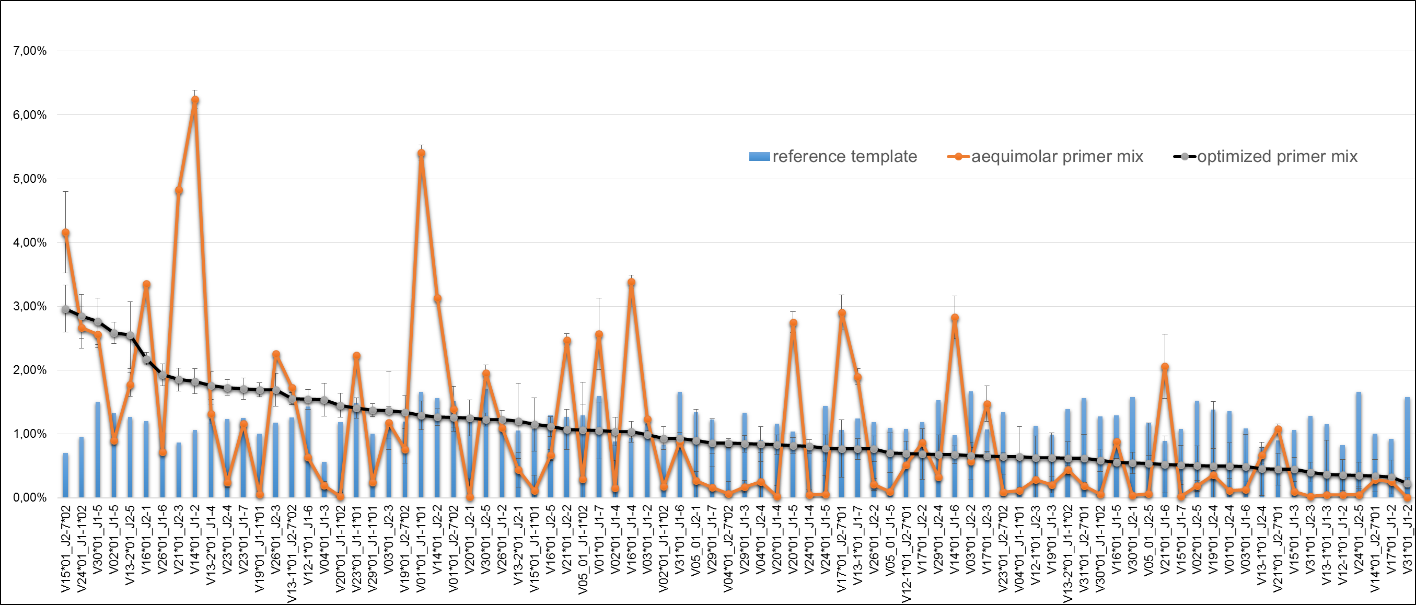


**Supplementary Figure S2**

**Optimization of TCRβ multiplex primer concentrations.** The quantities of the reference template sequences with their TCRβ V and J segment permutations are given (blue bars, mean 1.19 +/- 0.25 %, summing up for the 84 reference templates to 100%) as analyzed by TCRβ NGS. Furthermore the mean and standard deviation of 15 experiments analyzed by NGS for an equimolar TCRβ primer mix (orange line) in comparison to the TCRβ primer mix after 5 optimization rounds (black line) is shown.

**
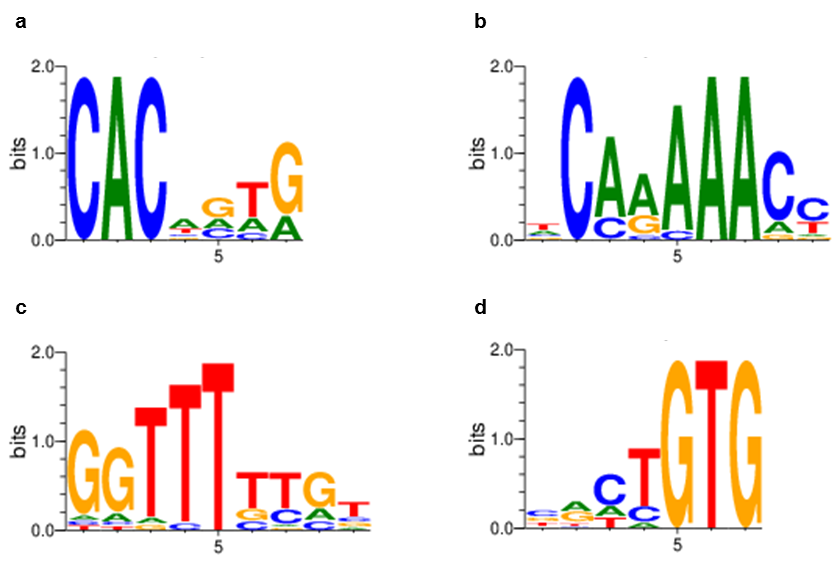
**

**Supplementary Figure S3**

**Sequence logos for generated heptamer and nonamer matrices. (a)** 3’ D segment heptamer. **(b)** 3’ D segment nonamer. **(c)** 5’ J segment nonamer. **(d)** 5’ J segment heptamer. Heights of nucleotide symbols correspond to relative frequencies within a given position. Heights of symbol stacks denote conservation and information content.

**Supplementary Tables**

**Supplementary Table S1: Statistics of TCRβ VN1DN2J architecture in wildtype, *Runx1^+/-^* and *Runx1^-/-^* mice thymus and spleen tissues.** Provided as Excel table.

**Supplementary Table S2: RUNX1 motif enrichment results.**

|  | All heptamer regions | Heptamer regions in RSS models |
| --- | --- | --- |
| Whole genome controls | | |
| Number of background heptamer hits | 4671973 | 167320 |
| Total length of background heptamer regions (bp) | 96558825 | 3509988 |
| RUNX1 motif matches in background heptamers  (+/- 7bp = 21 bp heptamer region)Kö | 1390814 | 42096 |
| Recurrent deletion borders in *ETV6-RUNX1* ALL | | |
| Number of deletion border heptamer hits | 65 | 15 |
| Total length of deletion border heptamer regions (bp) | 1358 | 315 |
| Expected RUNX1 motif match number in deletion border heptamer regions | 19.56 ± 4.39 SD | 3.78 ± 1.93 SD |
| Observed RUNX1 motif match number in deletion border heptamer regions | 46 | 14 |
| Enrichment | 2.35 | 3.71 |
| Z-score | 5.91 | 5.03 |
| *P* value (Binomial distribution) | *P* < 0.0000001 | *P* < 0.00004 |
| ChIP-Seq peaks of the *ETV6-RUNX1* ALL cell line REH | | |
| Number of ChiP-Seq peaks with RSS modules | not analyzed | 30 |
| Total length ChIP-Seq peak heptamer regions (bp) within RSS modules | not analyzed | 630 |
| Expected RUNX1 motif match number in ChIP-Seq peak heptamer regions with RSS modules | not analyzed | 7.56 ± 2.73 SD |
| Observed RUNX1 motif match number in ChIP-Seq peak heptamer regions within RSS modules | not analyzed | 15 |
| Enrichment | not analyzed | 1.99 |
| Z-score | not analyzed | 2.54 |
| *P* value (Binomial distribution) | not analyzed | *P* = 0.0105 |

**Supplementary Table S3: Genomic location of 56 reanalyzed *ETV6-RUNX1* deletion borders and the associated heptamer, nonamer and RUNX1 motif hits determined with the Genomatix Software Suite.** Provided as Excel table.

**Supplementary Table S4: TCRβ rearrangements in *RUNX1* mutated and *RUNX1* wildtype human T-ALLs.** Provided as Excel table.

**Supplementary Table S5: Primers used in this study to amplify murine TCRβ rearrangements.**

| Primer name | Primer sequence |
| --- | --- |
| MV1 | ggagctgaggctgcaagtggcc |
| MV2 | catttagaccttcagatcacagctct |
| MV3 | tatttcactctgaaaatccaacccaca |
| MV4 | ctcatttgaatcttcgaatcaagtctg |
| MV5 | ccagacagctccaagctacttttac |
| MV12-1/-2 | cactctgaaatgaacatgagtgcctt |
| MV13-1 | tcttcctcctgctggaattggct |
| MV13-2/-3 | tctccctcattctggagttggct |
| MV14 | ctccactctcaagatccagtctgca |
| MV15 | actctgaagattcaacctacagaac |
| MV16 | caactctgaagatccagagcacgca |
| MV17 | ctgctctctctacattggctctgca |
| MV19 | tctttttctctcactgtgacatctgc |
| MV20 | ccaacttatccttttcatctatgacagtt |
| MV21 | catgtaccatagagatccagtccagc |
| MV23 | tgcagcctgggaatcagaacgtgc |
| MV24 | catcctggaaatcctatcctctg |
| MV26 | gcagcctagaaattcagtcctctg |
| MV29 | catttctccctgattctggattctgc |
| MV30 | attctcaacgttgacagtgaacaatg |
| MV31 | ttcatcctaagcacggagaagctg |
| MJ1-1*01 | TTACCTACAACTGTGAGTCTGGTTC |
| MJ1-1*02 | TTACCTACAACTGTGAGTGTGGTTC |
| MJ1-2 | CTTACCTATTACCAAAAGCCTGGT |
| MJ1-3 | TACCTACAACAATGAGCCGGCTTCC |
| MJ1-4*01/*02 | TACCCAAGACAGACAGCTTGGTT |
| MJ1-5 | AGTTTACCTAGAACAGAGAGTCGAGT |
| MJ1-6 | CCTGTCACAGTGAGCCGGGTGC |
| MJ1-7 | GTCTTATCTTATACCTAAGTTCCTTTCCAA |
| MJ2-1 | TACCTAGGACGGTGAGTCGTGTC |
| MJ2-2 | ACCCAGCACTGTCAGCTTTGAGCC |
| MJ2-3 | TACCGAGAACAGTCAGTCTGGTTC |
| MJ2-4 | CCTAGCACCGATAGTCGGGTGC |
| MJ2-5 | CCTAACACGAGGAGCCGAGTGC |
| MJ2-7*01 | TACCTAAAACCGTGAGCCTGGTGC |
| MJ2-7*02 | TACCTAAAACCGTGAGCCTAGTGC |

21 forward primers and 15 reverse primers that anneal to the alleles of 23 known functional TCRβ V segments and 13 functional J segments respectively (http://www.imgt.org/).

**Supplementary Table S6: Primers used in this study to evaluate the Runx1 ChIP analysis.**

| Primer name | Primer sequence |
| --- | --- |
| Rag1_up | TCTGTGATTGGCTATCATCATCTGT |
| Rag1_low | TCTCACTGCCCTTCCCACTCT |
| TCRβ_D1_up | TGGTTTCTTCCAGCCCTCAA |
| TCRβ_D1_low | CCCACAATGTTACAGCTTTATACAAAA |
| TCRβ_D2_up | CCCCTCTCAGTCAGACAAACCT |
| TCRβ_D2_low | AAGCACCTCTTCCAGTTGAATCA |
| CD19_up | TGTGACCACCCCTTTCCTCTATAC |
| CD19_low | GCCTAGTGGGCAATGAACCA |
| Prame_up | CTGCTATTCTGCCAGCACTATCTAA |
| Prame_low | GGAGCATAGGCATTGAAATGG |

**Supplementary Table S7: Heptamer and nonamer sequences of 3´ TCRβ D segments, 3´ TCRδ D segments, 3´ IgH D segments, 5´ TCRβ J segments, 5´ TCRδ J segment, 5´ IgH J segments according to IMGT annotations.**

| Heptamer | Frequency | Nonamer | Frequency |
| --- | --- | --- | --- |
| 3´ TCRβ D segment heptamer and nonamer sequences | | | |
| CACGATG | 1 | ACAAAAACC | 1 |
| CACAATG | 1 | ACAAAAAAC | 1 |
| 3´ TCRδ D segment heptamer and nonamer sequences | | | |
| CACACAG | 1 | CCAAAAACA | 1 |
| CACTCAA | 1 | ATTAACCAA | 1 |
| CACAGTG | 1 | ACAAAAACT | 1 |
| 3´ IgH D segment heptamer and nonamer sequences | | | |
| CACAGCA | 1 | TCAAAAACT | 1 |
| CACAATG | 1 | TCAAAAACC | 3 |
| CACCGTG | 2 | CCAGAAACC | 3 |
| CACTGTG | 2 | TCCAAAACT | 3 |
| CACAGTG | 17 | ACAAAAACC | 1 |
|  |  | TCAGAAAAC | 1 |
|  |  | TCCCAAAGC | 1 |
|  |  | ACAGAAACC | 1 |
|  |  | GCAAAAACT | 2 |
|  |  | TCCAAAACG | 1 |
|  |  | GCAGCAACC | 3 |
| 5´ TCRβ J segment heptamer and nonamer sequences | | | |
| GAATTCTGG | 1 | CTCCGTG | 1 |
| CCTTTTAGA | 1 | GGCTGTG | 5 |
| GGTTTTTGT | 2 | AGCTGTG | 1 |
| GGTTTTGAA | 1 | TTATGTG | 1 |
| GGTTTGCAT | 1 | CACTGTG | 3 |
| GATTTTCAC | 1 | GGCCGTG | 1 |
| AGTTTCTGT | 1 | TGTTGTG | 1 |
| GGGTTTGCC | 1 |  |  |
| GGTTTTTGC | 1 |  |  |
| GGTTTTCCT | 1 |  |  |
| GGTTTGCGC | 1 |  |  |
| GGGTTTTAT | 1 |  |  |
| 5´ TCRδ J segment heptamer and nonamer sequences | | | |
| GGTTTTTCG | 1 | TGCTGTG | 1 |
| GGTTTTTGG | 1 | TAATGTG | 1 |
| GTTACCTGT | 1 | GGTAGTG | 1 |
| 5´ IgH J segment heptamer and nonamer sequences | | | |
| GGTTTTTGT | 2 | CAATGTG | 2 |
| GGTTTGTGT | 1 | CCCTGTG | 1 |
| GGTTTCTGT | 1 | GACTGTG | 1 |
| GTTCTTTGT | 1 | CATTGTG | 1 |
| TGTTTTTGT | 1 | CACCGTG | 1 |

**Supplementary Table S8: RSS model definitions.**

|  | Matrix 1 | Strand | Threshold | Spacer | Matrix 2 | Strand | Threshold |
| --- | --- | --- | --- | --- | --- | --- | --- |
| Model 1 | 3’ D Hept | + | 0.98 | 11-13 nt | 3’ D Nona | + | 0.84 |
| Model 2 | 3’ D Hept | + | 0.98 | 22-24 nt | 3’ D Nona | + | 0.84 |
| Model 3 | 5’ J Nona | + | 0.81 | 11-13 nt | 5’ J Hept | + | 0.98 |
| Model 4 | 5’ J Nona | + | 0.81 | 22-24 nt | 5’ J Hept | + | 0.98 |
| Model 5 | 5’ J Hept | - | 0.98 | 11-13 nt | 3’ D Nona | + | 0.84 |
| Model 6 | 5’ J Hept | - | 0.98 | 22-24 nt | 3’ D Nona | + | 0.84 |
| Model 7 | 5’ J Nona | + | 0.81 | 11-13 nt | 3’ D Hept | - | 0.98 |
| Model 8 | 5’ J Nona | + | 0.81 | 22-24 nt | 3’ D Hept | - | 0.98 |

Threshold: matrix similarity threshold used in the search for matches; higher thresholds mean higher stringency. Nonamers have compared to heptamers more variability in the degree to which individual positions are conserved, therefore a lower threshold was employed for nonamers^1^. Strand: matrix strand orientation.

**Literature**

1 Ramsden, D. A., Baetz, K. & Wu, G. E. Conservation of sequence in recombination signal sequence spacers. *Nucleic Acids Res* **22**, 1785-1796 (1994).
